# Supplementary material for: Zinc is a master-regulator of sperm function associated with binding, motility, and metabolic modulation during porcine sperm capacitation
Source: Commun Biol. 2022 Jun 3;5:538. doi: 10.1038/s42003-022-03485-8 (PMC9166710; doi:10.1038/s42003-022-03485-8)
Supplement: Supplementary file 2 — Supplementary Information [file 42003_2022_3485_MOESM2_ESM.pdf]

Supplementary Information for

Zinc is a master-regulator of sperm function associated with binding, motility, and metabolic modulation during porcine sperm capacitation

Michal Zigo, Karl Kerns, Sidharth Sen, Clement Essien, Richard Oko, Dong Xu, and Peter Sutovsky

Correspondence to: zigom@missouri.edu, alt. michal_zigo_2000@yahoo.com; SutovskyP@missouri.edu

This PDF file includes:

Supplementary Discussion
Figs. S1 to S16

Other Supplementary Materials for this manuscript include the following:

Supplementary Data 1 – All zinc-binding proteins with UniProtKB accessions, spectral counts, normalization, and stats
Supplementary Data 2 – Prediction of zinc-binding sites data
Supplementary Data 3 – Panther analysis data, volcano plot data
Supplementary Data 4 – Pie charts source data
Supplementary Data 5 – Significantly different zincoproteins + functions + localization + references

Supplementary Discussion - Implications of the three more closely studied zincoprotein

This section is focused on the implications of the three more closely studied zincoprotein that were found significantly different in abundance between non-capacitated (ejaculated) and IVC spermatozoa, namely, carbonic anhydrase 2 (CA2), calicin (CCIN), and coiled-coil domain containing 39 (CCDC39). Carbonic anhydrases together with HCO_3^- influx system are two sperm systems responsible for pH and HCO_3^- homeostasis; reviewed in[1]. pH and HCO_3^- homeostasis is needed for spermatozoa to capacitate, be motile, and hyperactivated. There are at least 15 known carbonic anhydrase isoenzymes with 12 catalytic active isoforms[2], however, it has been demonstrated that CA1, CA2, and CA13 are present in human spermatozoa while isoforms CA2, CA4, and CA12 are found in mouse spermatozoa[3]. We have identified CA2 and CA3 in boar zincoproteome in this study, with CA2 being the more abundant one. CA2 in boar spermatozoa immunolocalizes to the sperm head acrosomal region, and the sperm tail midpiece and principal piece. The CA2 localization seems to differ among species; CA2 was found to localize to the acrosome and the flagellum in mice[3,4], in the acrosomal cap region of the rat spermatozoa[5], and the post-acrosomal region and the flagellum of the human spermatozoa[3,5]. Little is known about the potential role of CA2 in sperm capacitation. We observed a decrease in the abundance of CA2 after IVC, by both IBFC (fig. S4) and WB (fig. S5), and a localized fluorescence intensity decreased in the midpiece by both ICC and IBFC. The functional implication of capacitation-related shedding of CA2 from the midpiece region is subject to further study. Mice knockout studies[4] revealed that $CA2^{-/-}$ and $CA4^{-/-}$ single knockouts displayed an imbalanced HCO_3^- homeostasis resulting in substantially reduced sperm motility, swimming speed, and HCO_3^--enhanced beat frequency. A comparative study of carbonic anhydrases between human and mouse was performed[3]. The key findings were that capacitated human spermatozoa strongly depend on CA activity to support normal motility, while capacitated mouse spermatozoa do not.

Furthermore, the inhibition of CA activity in capacitated human spermatozoa increases the acrosomal exocytosis, but such an increase is not observed in capacitated mouse spermatozoa. In a recent review by Di Fiore et al.[6], an extensive literature search was performed to summarize post-translational modifications of human carbonic anhydrases. There is a widespread occurrence of modification events on all CAs often detected at conserved amino acids, including phosphorylation, non-enzymatic glycation, N-glycosylation, O-glycosylation, S-glutathionylation, S-nitrosylation, disulfide-formation, acetylation, ubiquitination, methylation, and GPI-anchoring. Despite the clear correlation between a specific PTM and the CA function in some cases, further dedicated studies will be required to elucidate the biological relevance of these modifications.

Calicin (CCIN) is a major basic protein of the mammalian head sperm cytoskeleton that was first reported as a component of bull perinuclear theca (PT)[7]. More attention was given to CCIN by Oko and Maravei[8] where the authors extracted PT proteins from the acrosome-free bull sperm heads. Among the extracted proteins, CCIN migrated at 60 kDa (hence the initial PT60 designation) and localized to both the subacrosomal layer and postacrosomal sheath of PT. The same authors showed that CCIN is engaged in the development of the acrosomic system early in spermiogenesis; and after the acrosome had capped the nucleus, the labeling resided in the subacrosomal layer of the nucleus[9]. Later, during the spermatid elongation, CCIN extended caudally over the nascent postacrosomal sheath. During spermiogenesis, CCIN associates with F-actin with high affinity and is closely tied to the acrosomal formation[10]. For more in-depth reviews of PT formation during spermatogenesis, we recommend[11,12]. cDNA cloning of CCIN revealed that it contains a region of ~100 amino acids homologous to an extended motif shared by the kelch protein family as well as various zinc finger motifs[13].

In the present study, we focused on the fate of CCIN, as one of the identified zincoproteins, during boar sperm capacitation. We immunolocalized CCIN in the postacrosomal region of ejaculated (non-capacitated) spermatozoa and the whole sperm head in IVC spermatozoa. We speculate that similarly as in bull spermatozoa, the CCIN is present in both the subacrosomal layer and postacrosomal sheath; however, the subacrosomal layer is inaccessible to the anti-CCIN antibody in ejaculated spermatozoa. During capacitation, a spermatozoon is subjected to biophysical and biochemical changes that prepare it for fertilization, Zn^{2+} efflux being one of them[14] that might loosen up PT, making it more accessible for antibody labeling in the subacrosomal region. We also identified other known PT proteins within the zincoproteome, such as RAB2A and H2B. As a result of capacitation, these proteins are detected in higher abundances; for CCIN $P=0.039$, for RAB2A $P=0.077$ (Tab S1). The CCIN extractability increase in capacitated spermatozoa was also confirmed by WB of the alkali and SDS extracts (fig. S10). Earlier, we reported a posterior-to-anterior plasma membrane and nuclear envelope modification wave that was reflected by incorporation of propidium iodide, a fluorescent tracer of increased membrane permeability, to the nuclei of live, capacitating spermatozoa in the same direction[15]. We have observed the same trend with the anti-CCIN antibody accessibility as a posterior-to-anterior wave of the CCIN fluorescence signal detection that is reflected as the presence of sperm populations with different fluorescence signal intensity in IVC spermatozoa (fig. S6). It can be hypothesized that in addition to the plasma membrane and nuclear envelope modification during sperm capacitation, it is the PT loosening as a result of organizational changes of PT proteins that facilitates the PI incorporation into the nucleus. Whichever the case is, we can agree that CCIN as well as other PT proteins have a very important structural and regulatory role during sperm capacitation that has been overlooked.

The last of the three studied zincoprotein, CCDC39 is important for the proper assembly of inner dynein arms of cilia and flagella and the mutation of the *CCDC39* gene is a cause of primary ciliary dyskinesia [[16,17], reviewed in[18]]. The observed phenotype of men and dogs with *CCDC39* mutation is microtubular disorganization that resulted in oligoasthenospermia[16,17]. In the present study, we found CCDC39 to be significantly more abundant in the capacitated sperm zincoproteome when compared to ejaculated one. We found CCDC39 to immunolocalize in the flagellum and the apical ridge of the acrosomal region in boar spermatozoa. While the localization and function of CCDC39 in the flagellum is indisputable, to our best knowledge, neither the localization nor the function of CCDC39 in the sperm head has been reported previously. We can speculate from the CCDC39 localization that it might play a role in the acrosomal integrity of the spermatozoon, and/or be a vestige of microtubule involvement in acrosomal biogenesis during spermatid differentiation. Interestingly, the acrosomal CCDC39 localization is shifted from a relatively wider area in ejaculated spermatozoa to a narrower region in IVC spermatozoa that is reflected by a decrease of fluorescence intensity signal monitored by IBFC (fig. S11). CCDC39 in ejaculated and IVC sperm zincoproteome was below the limit of detection when we performed WB analysis (fig. S12); however, the CCDC39 abundance level was roughly the same in the whole sperm extract of ejaculated and IVC spermatozoa (Extended Data Fig. 2I). Judging from its dual localization, it seems that CCDC39 may play multiple roles in boar spermatozoa. It has been reported that CCDC39 is required for the correct assembly of inner dynein arms in the forming sperm flagellum during spermatogenesis[18], however, its presence in the mature sperm flagellum has not been fully clarified to this day. Additional studies are required to elucidate the function of acrosomal CCDC39 in sperm capacitation.

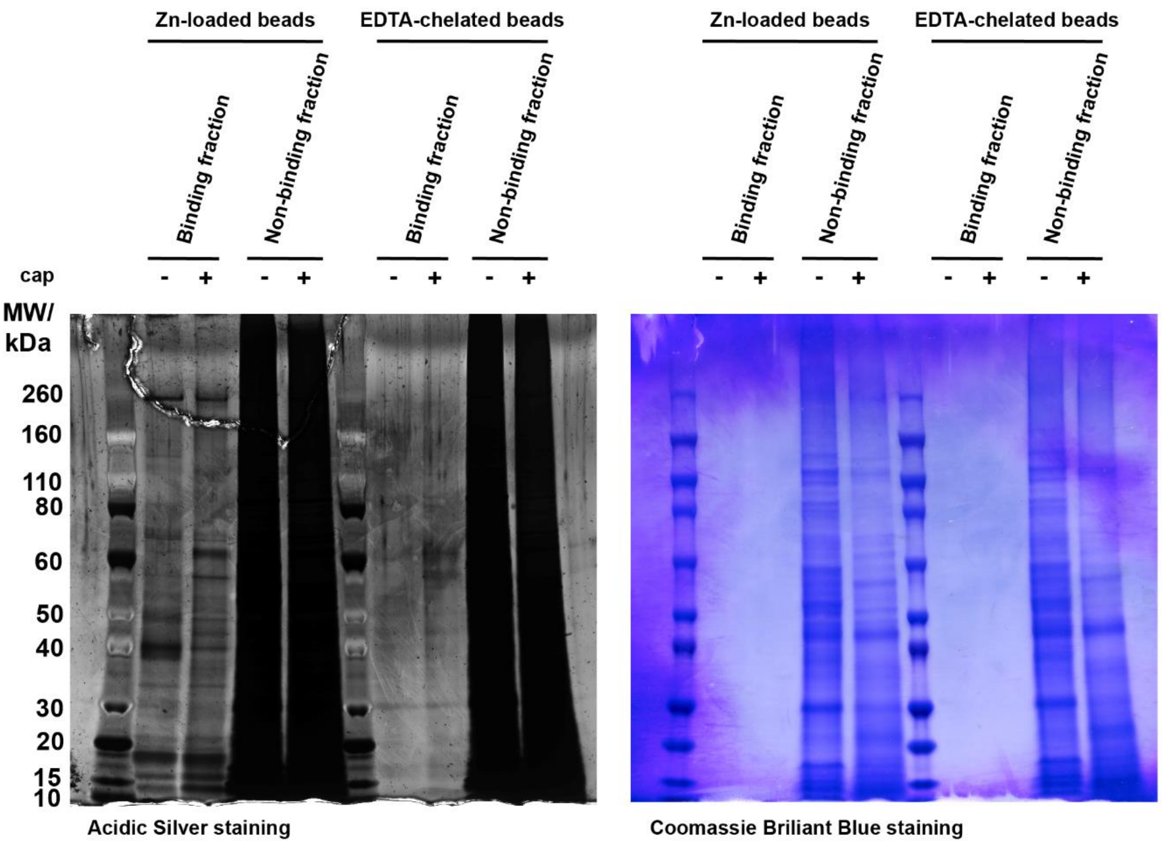

Fig. S1.

Proteins bound to the Zn^{2+}-immobilized beads abundantly, while only limited binding was observed with EDTA-stripped beads. To test the specificity of Immobilized Metal Ion Affinity chromatography, the extracts from ejaculated and IVC spermatozoa were incubated with Zn^{2+} immobilized beads or EDTA-chelated beads. The Zn^{2+} immobilized and EDTA-chelated beads' binding and non-binding fractions were resolved by 1-DE on 4-12 % Bis-Tris gel, stained with CBB, destained, and restained with acidic silver staining (cat# 1610449, Bio-Rad).

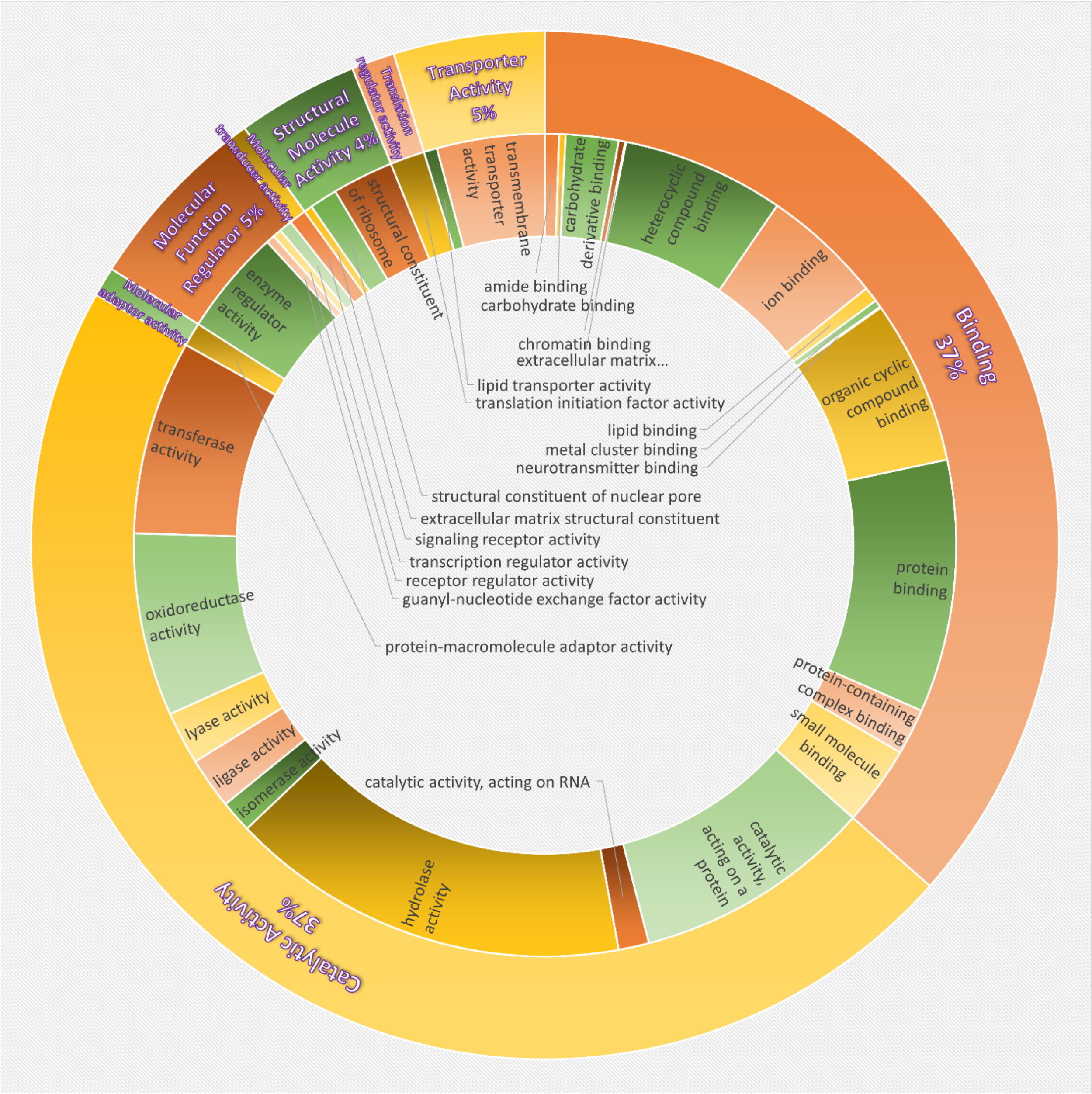

Fig. S2.

PANTHER molecular function analysis of identified zincoproteins. The outer circle represents high level (level 0), while sub-level (level 1) is represented by the inner circle of the respective doughnut charts. For clarity, some of the level 1 categories were removed; all categories are shown in Table S2.

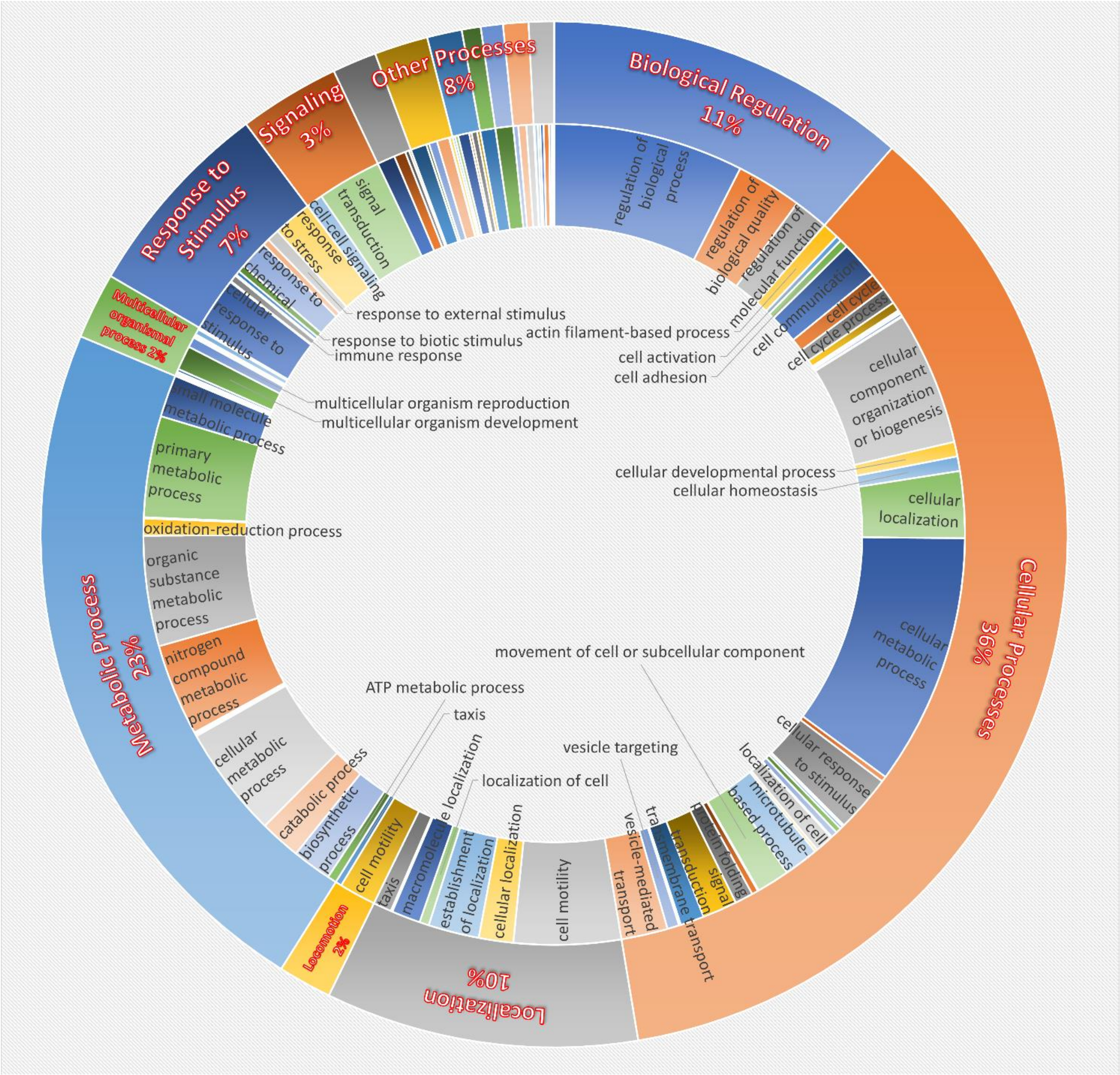

Fig. S3.

PANTHER biological process analysis of identified zincoproteins. The outer circle represents high level (level 0), while sub-level (level 1) is represented by the inner circle of the respective doughnut charts. For clarity, the chart displays the first eight most abundant categories (cumulatively 92%); and some of the level 1 categories were removed as well; all categories for both levels are shown in Table S2.

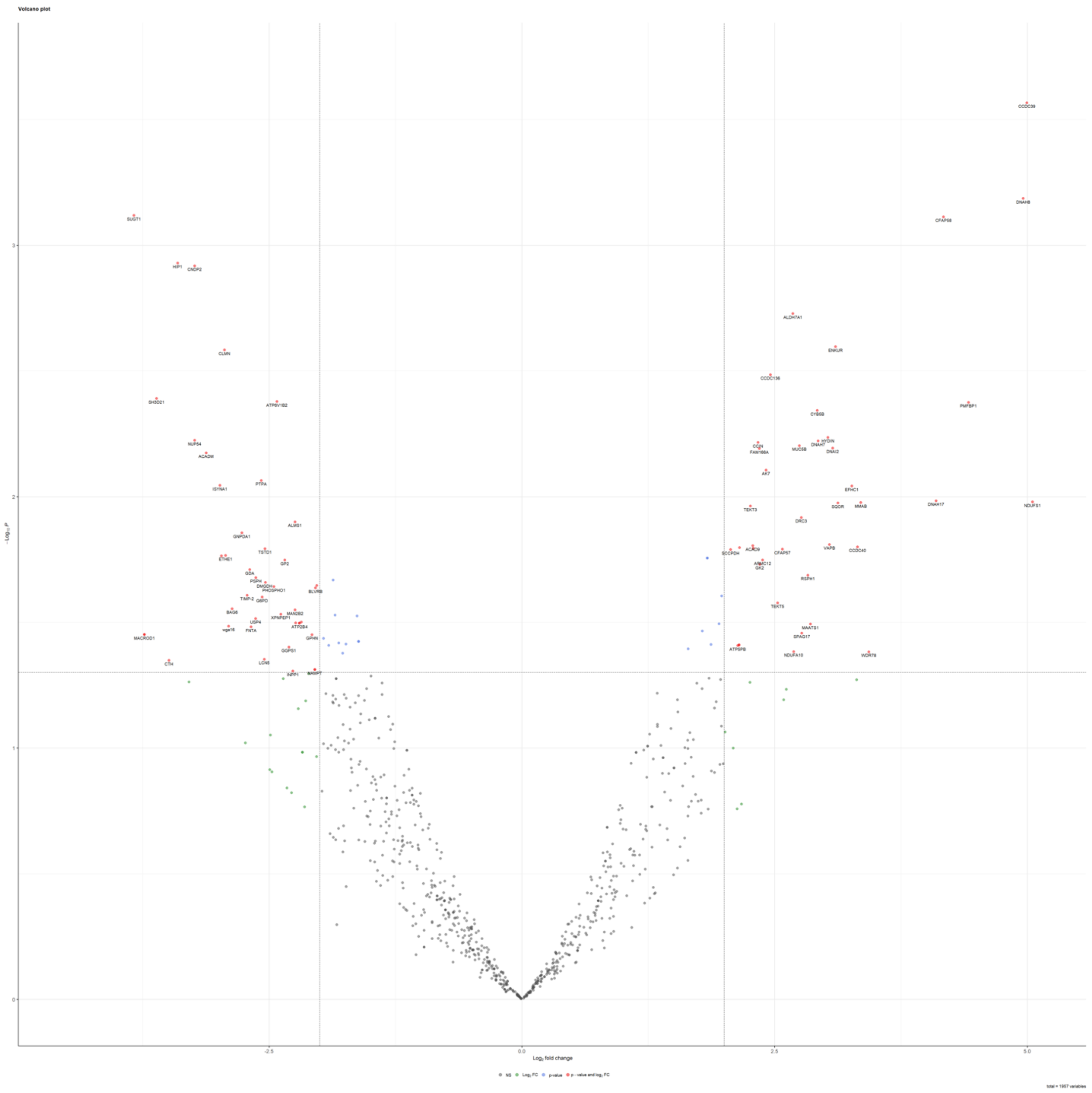

Fig. S4.

High-resolution volcano plot analysis from Fig 3 represents changes in zincoprotein abundances before and after 4 hours of in vitro capacitation. Fold change threshold = 2.0 (x-axis) and unadjusted P-value ≤ 0.05 (y-axis). Red circles represent zincoproteins above the fold change and unadjusted P-value threshold; green circles represent those above the unadjusted P-value threshold but below the fold change threshold; blue circles represent zincoproteins above the fold change but below the unadjusted P-value threshold; and gray circles represent those that are below both thresholds.

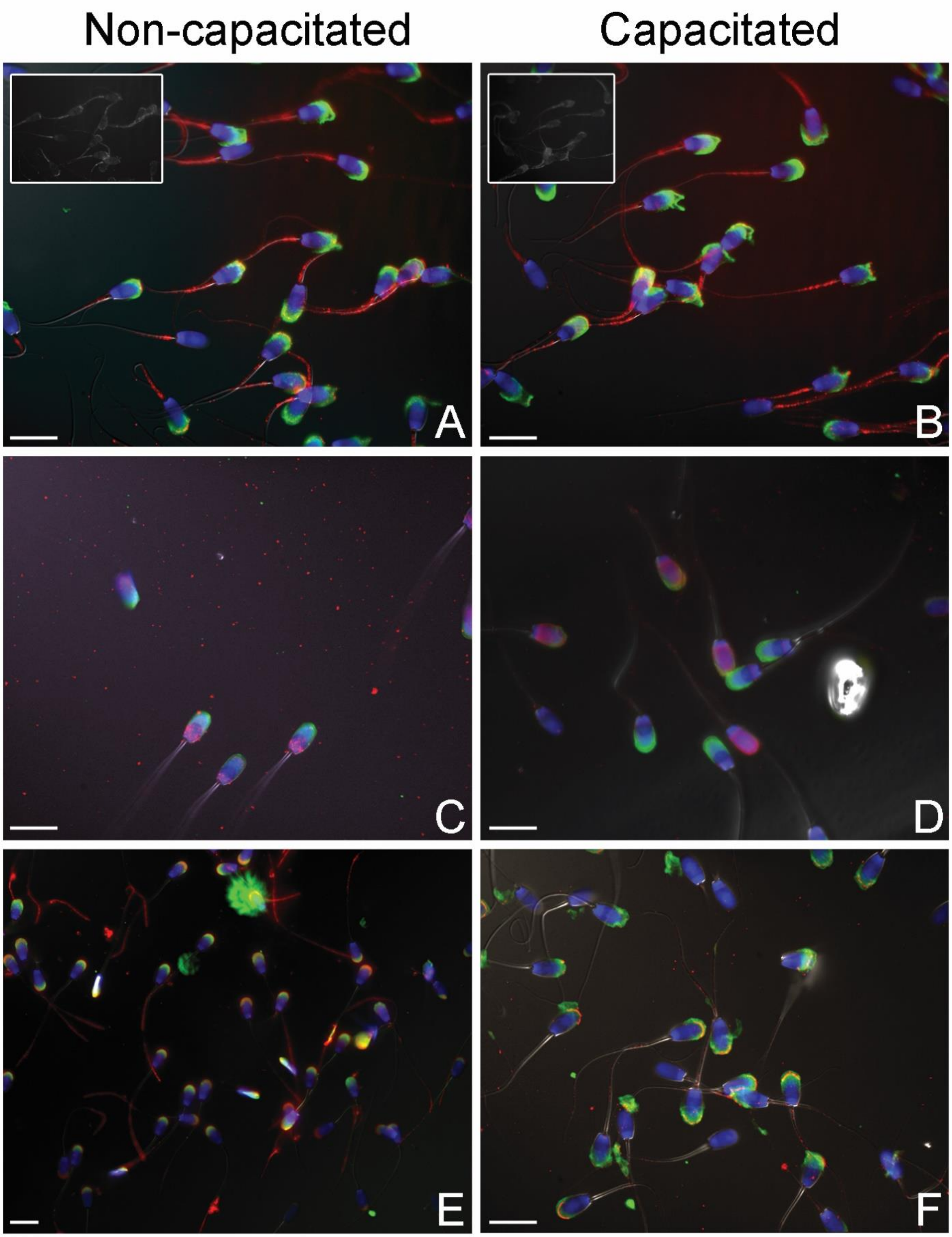
Non-capacitated
Capacitated
A
B
C
D
E
F

Fig. S5.

Immunolocalization of the three zincoproteins (red pseudocolor) in fixed and permeabilized spermatozoa that were significantly different between ejaculated and IVC spermatozoa. CA2 localizes in acrosomes and flagella in both ejaculated (A) and IVC spermatozoa (B); the insets show CA2 channel only. CCIN immunofluorescence signal was detected in the post-acrosomal segment of ejaculated spermatozoa (C) and the whole area of perinuclear theca (subacrosomal, equatorial & post-acrosomal) of IVC spermatozoa (D). CCDC39 localizes to the principal piece and apical ridge of sperm head in both ejaculated (E) and IVC (F) spermatozoa. Spermatozoa were co-stained for acrosomal integrity with peanut agglutinin lectin (PNA, green), and a nuclear stain DAPI (blue). All fluorescence channels are superimposed with the DIC brightfield channel. Scale bars represent 10 μm.

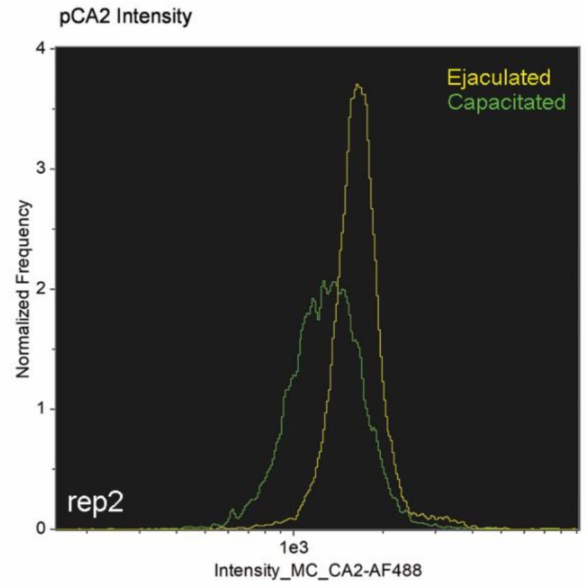

Intensity_MC_CA2-AF488

Population	Count	Objects/mL	Mean	Median	Std. Dev.	Mode
Non-Capac Spermatozoa	8514	1257474.31	1688.96	1638.61	486.29	1638.58
Capacitated Spermatozoa	7408	1094123.76	1340.32	1291.08	514.99	1170.58

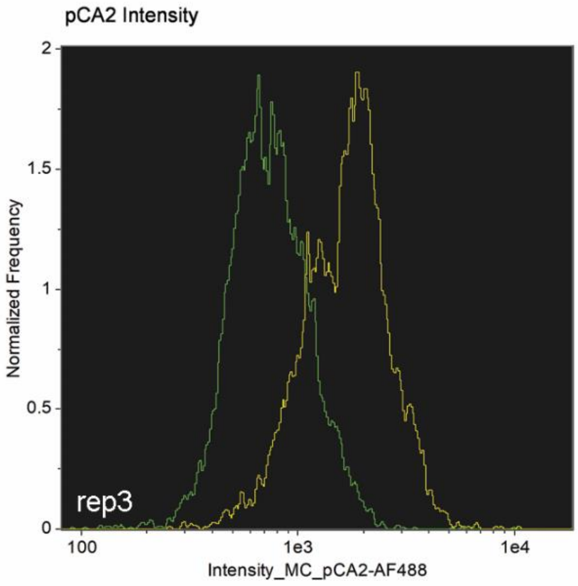

Intensity_MC_pCA2-AF488

Population	Count	Objects/mL	Mean	Median	Std. Dev.	Mode
Capacitated Spermatozoa	4429	371027.9	805.69	726.43	388.73	637.74
Non-Capac Spermatozoa	3498	293035.81	1828.88	1739.91	880.55	1842.72

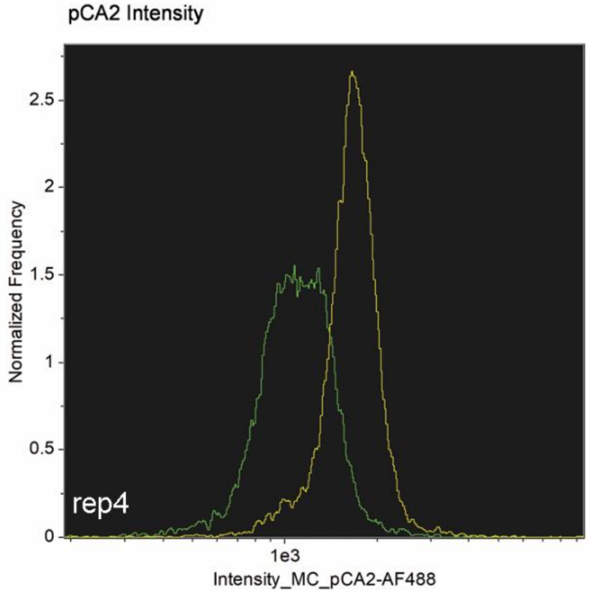

Intensity_MC_pCA2-AF488

Population	Count	Objects/mL	Mean	Median	Std. Dev.	Mode
Capacitated spermatozoa	7599	636586.36	1129.56	1093.28	358.16	997.36
Non-Capac spermatozoa	9401	787544.2	1683.31	1668.39	419.66	1685.4

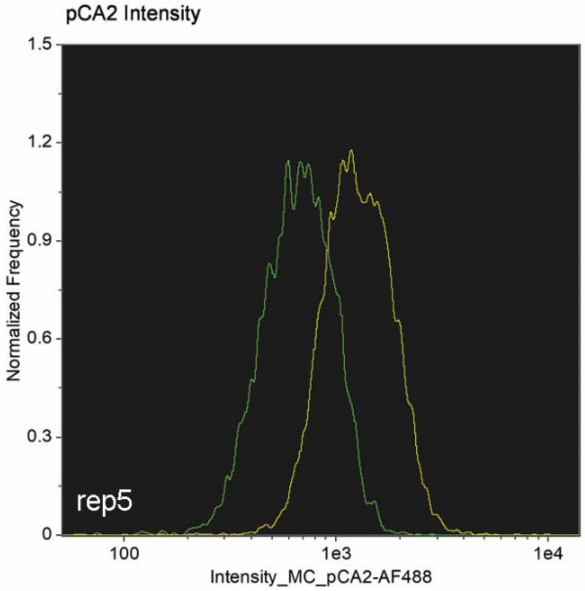

Intensity_MC_pCA2-AF488

Population	Count	Objects/mL	Mean	Median	Std. Dev.	Mode
Capacitated Spermatozoa	5804	168402.13	711.14	672.74	295.46	592.33
Non-Capac Spermatozoa	6480	188016.16	1358.1	1260.86	523.01	1059.98

Fig. S6.

The remaining four replicates of capacitation-related decrease of CA2 fluorescence intensity, complementing the one shown in Fig 6A, with the corresponding descriptive statistics.

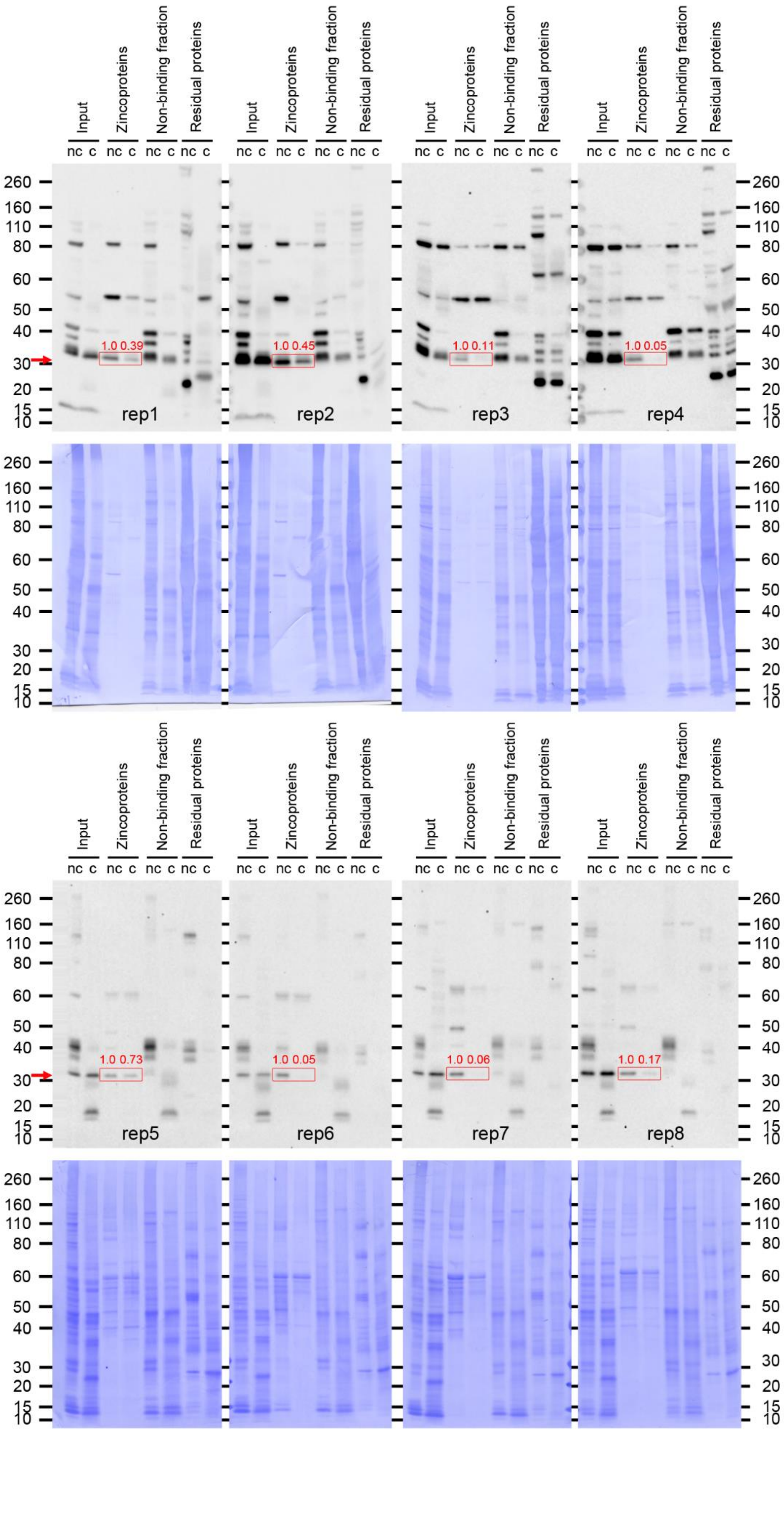

Input
Zincoproteins
Non-binding fraction
Residual proteins
nc c nc c nc c nc c
260
160
110
80
60
50
40
30
20
15
10
1.0 0.39
1.0 0.45
1.0 0.11
1.0 0.05
rep1
rep2
rep3
rep4
1.0 0.73
1.0 0.05
1.0 0.06
1.0 0.17
rep5
rep6
rep7
rep8

Fig. S7.

All eight replicates of CA2 WB detection from Fig 6B show a CA2 decrease in the sperm zincoproteome after *in vitro* capacitation. The red arrows point to the bands of actual CA2 ~ 32 kDa. Red rectangles show CA2 pulled down in the zincoproteome fractions with relative band abundances. The intensity of the CA2 bands in non-capacitated zincoproteome fractions was set to 1.00 as default and the relative intensity of the bands in capacitated zincoproteome fractions are related to their respective non-capacitated control. Membranes were stained with the CBB after the chemiluminescence detection for protein loading control (~ same protein load between non-capacitated and capacitated samples within the respective groups) and normalization purposes. The corresponding CBB stained membrane is depicted under its WB detection complement.

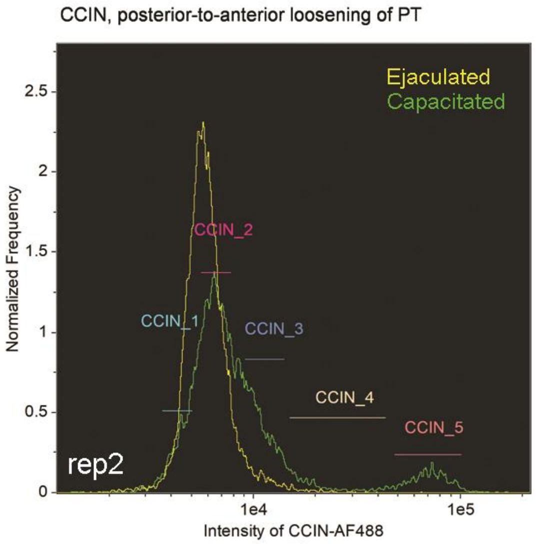

Intensity of CCIN-AF488

Population	Count	%Gated	Mean	Median	Std. Dev.	Mode
Capacitated Spermatozoa	8513	100	12461.75	7184.62	17431.87	6394.3
CCIN_1 & Capacitated Sperm...	995	11.7	4515.03	4540.8	394.14	4957.21
CCIN_2 & Capacitated Sperm...	3200	37.6	6585.37	6542.24	614.89	6556.83
CCIN_3 & Capacitated Sperm...	1552	18.2	10953.96	10636.04	1361.75	9242.86
CCIN_4 & Capacitated Sperm...	333	3.91	21752.5	18540.21	7610.13	15247.06
CCIN_5 & Capacitated Sperm...	534	6.27	72200.82	71885.58	12706.77	73050.79

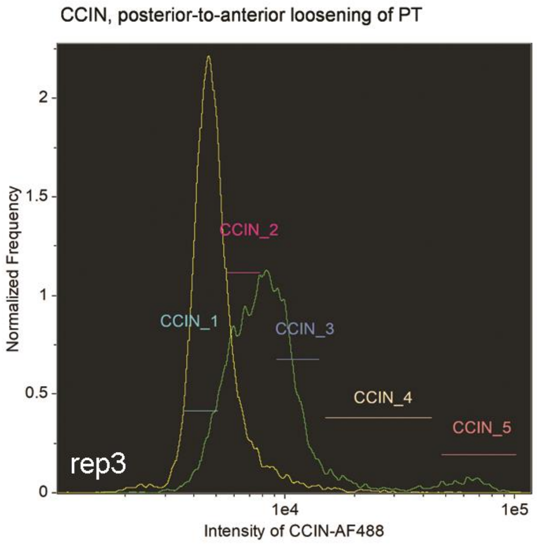

Intensity of CCIN-AF488

Population	Count	%Gated	Mean	Median	Std. Dev.	Mode
Capacitated Spermatozoa	7187	100	10653.41	8064.32	11283.99	8025.23
CCIN_1 & Capacitated Sperm...	559	7.78	4595.2	4681.38	369.17	4705.24
CCIN_2 & Capacitated Sperm...	2307	32.1	6674.19	6674.13	642.37	5813.76
CCIN_3 & Capacitated Sperm...	1897	26.4	10659.95	10343.77	1207.04	9222.58
CCIN_4 & Capacitated Sperm...	320	4.45	22437.68	19094.21	7799.01	15675.39
CCIN_5 & Capacitated Sperm...	234	3.26	64880.49	64204.89	10528.1	61871.62

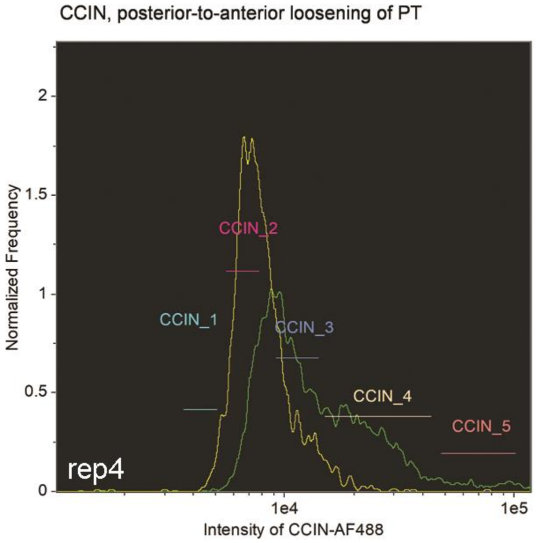

Intensity of CCIN-AF488

Population	Count	%Gated	Mean	Median	Std. Dev.	Mode
Capacitated Spermatozoa	4960	100	17418.88	11545.9	17607.86	8705.93
CCIN_1 & Capacitated Sperm...	5	0.1	4857.61	4916.79	220.56	4914.24
CCIN_2 & Capacitated Sperm...	616	12.4	7074.23	7161.57	503.49	7425.63
CCIN_3 & Capacitated Sperm...	1599	32.2	11081.5	10818.99	1397.48	9301.64
CCIN_4 & Capacitated Sperm...	1550	31.2	23383.94	21819.56	6588.94	15443.62
CCIN_5 & Capacitated Sperm...	151	3.04	70829.83	65901	16425.16	48505.07

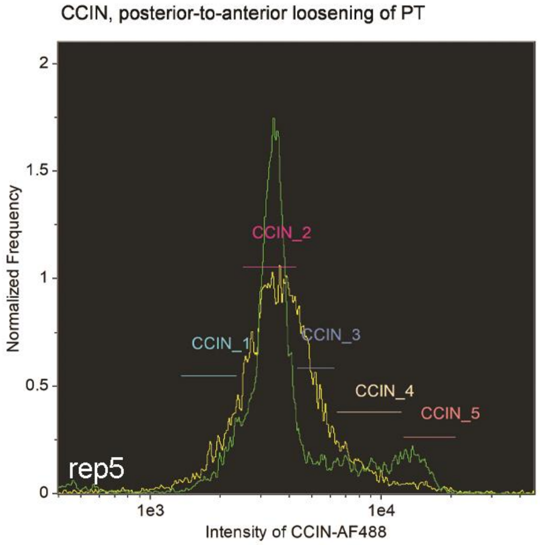

Intensity of CCIN-AF488

Population	Count	%Gated	Mean	Median	Std. Dev.	Mode
Capacitated Spermatozoa	9556	100	4190.93	3391.48	3570.93	3421.51
CCIN_1 & Capacitated Sperm...	614	6.43	2042.89	2105.72	248.03	2341.01
CCIN_2 & Capacitated Sperm...	5199	54.4	3383.17	3390.64	394.72	3449.51
CCIN_3 & Capacitated Sperm...	630	6.59	5117.52	5036.14	564.19	4371.58
CCIN_4 & Capacitated Sperm...	880	9.21	9062.18	8971.65	1753.74	6504.09
CCIN_5 & Capacitated Sperm...	547	5.72	14627.84	14331.95	1619.19	12750.23

Fig. S8.

The other four replicates of the capacitation-related posterior-to-anterior spread of the CCIN signal (from Fig 6D) gave rise to five different CCIN localization patterns. The corresponding descriptive statistics for CCIN populations in capacitated spermatozoa are presented as well.

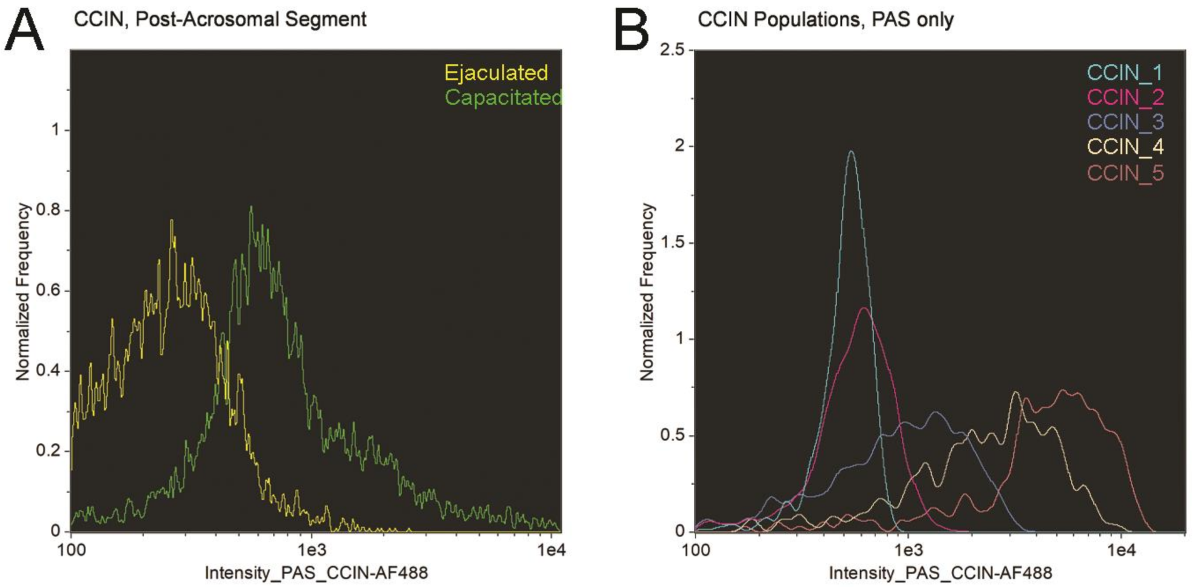

Intensity_PAS_CCIN-AF488

Population	Count	Objects/mL	Mean	Median	Std. Dev.	Mode
Non-Capac spermatozoa	4359	2063312.92	291.3	249.39	199.4	100.06
Capacitated spermatozoa	5822	2755817.34	1078.17	673.74	1252.41	540.99

Intensity_PAS_CCIN-AF488

Population	Count	Objects/mL	Mean	Median	Std. Dev.	Mode
CCIN_1 & Capacitated Sp	489	231465.94	521.79	529.47	125.61	510.93
CCIN_2 & Capacitated Sp	2516	1190937.21	603.55	591.64	230.59	630.74
CCIN_3 & Capacitated Sp	1591	753092.65	1089.15	967.25	678.98	223.39
CCIN_4 & Capacitated Sp	241	114076.26	2863.03	2534.28	1830.12	1192.26
CCIN_5 & Capacitated Sp	227	107449.42	5280.79	5031.91	2986.73	3384.28

Fig. S9.

Capacitation-related increase of the PAS fluorescence intensity (A), and a corresponding increase of PAS fluorescence intensity in CCIN subpopulations of capacitated spermatozoa (B), with corresponding descriptive statistics.

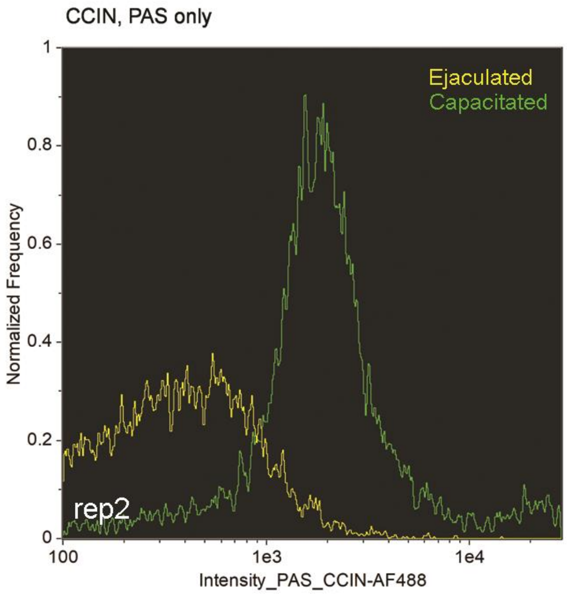

Intensity_PAS_CCIN-AF488

Population	Count	Objects/mL	Mean	Median	Std. Dev.	Mode
Non-Capac Spermatozoa	9396	1000464.59	339.49	177.75	640.23	237.78
Capacitated Spermatozoa	8513	906444.77	3373.48	1809.5	6118.29	1713.8

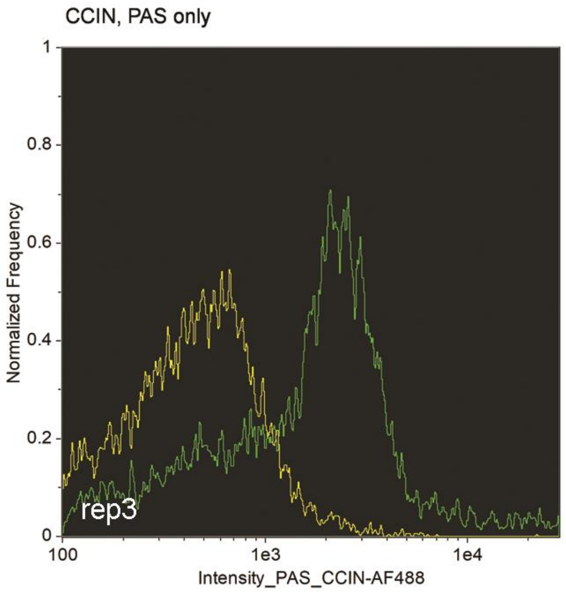

Intensity_PAS_CCIN-AF488

Population	Count	Objects/mL	Mean	Median	Std. Dev.	Mode
Non-Capac Spermatozoa	9093	3961587.13	438.53	330.12	639.11	209.3
Capacitated Spermatozoa	7187	3131191.77	2545.97	1720.43	4427.68	334.39

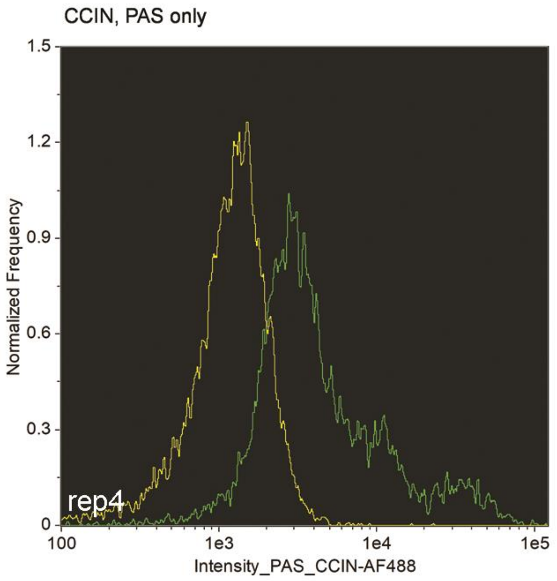

Intensity_PAS_CCIN-AF488

Population	Count	Objects/mL	Mean	Median	Std. Dev.	Mode
Non-Capac Spermatozoa	8485	3474294.2	1296.69	1227.39	765.09	1208.51
Capacitated Spermatozoa	6361	2604594.62	7378.25	3479.4	10768.96	2622.9

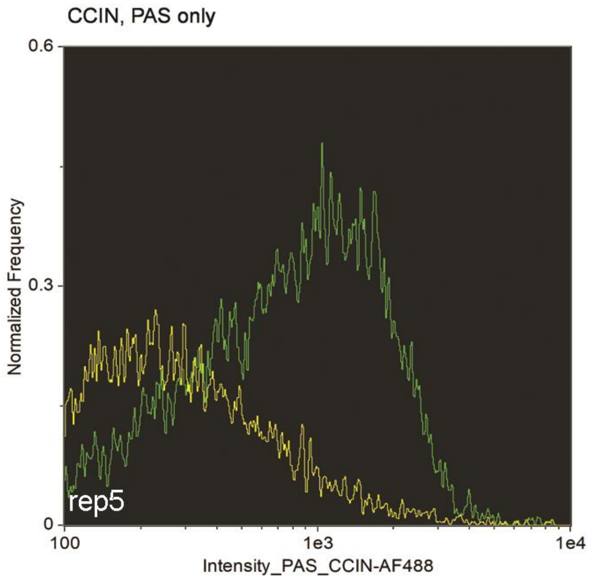

Intensity_PAS_CCIN-AF488

Population	Count	Objects/mL	Mean	Median	Std. Dev.	Mode
Non-Capac Spermatozoa	9556	2676740.74	224.14	77.18	499.31	81.11
Capacitated Spermatozoa	8602	2409514.85	942.84	705.45	1228.18	171.83

Fig. S10.

The other four replicates of the capacitation-related increase of the PAS fluorescence intensity from Fig S9A, with the corresponding descriptive statistics.

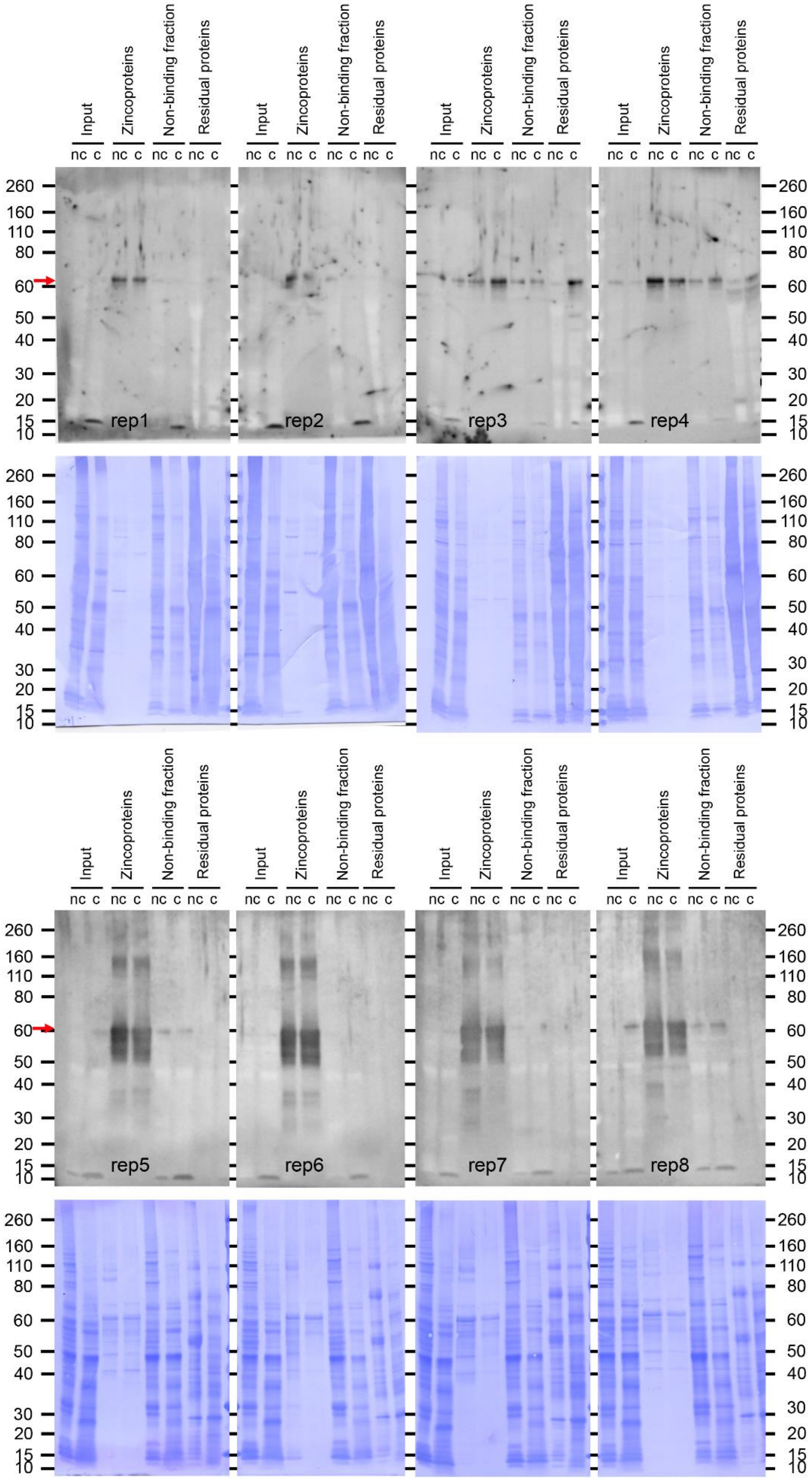
Input
Zincoproteins
Non-binding fraction
Residual proteins
nc c nc c nc c nc c
260
160
110
80
60
50
40
30
20
15
10
rep1
rep2
rep3
rep4
rep5
rep6
rep7
rep8

Fig. S11.

All eight replicates of CCIN WB detection from Fig 6E. Approximately 2.5x higher load of zincoproteins was used in replicates 5-8 resulting in more background in the zincoprotein fractions. For densitometric quantification, the intensity of the 60 kDa band was used consistently throughout all 8 replicates. Membranes were stained with the CBB after the chemiluminescence detection for protein loading control and normalization purposes. The corresponding CBB stained membrane is depicted under its WB detection complement. It is apparent from the CBB stained membrane that the total protein load of zincoproteins is higher in replicates 5-8 when compared to 1-4.

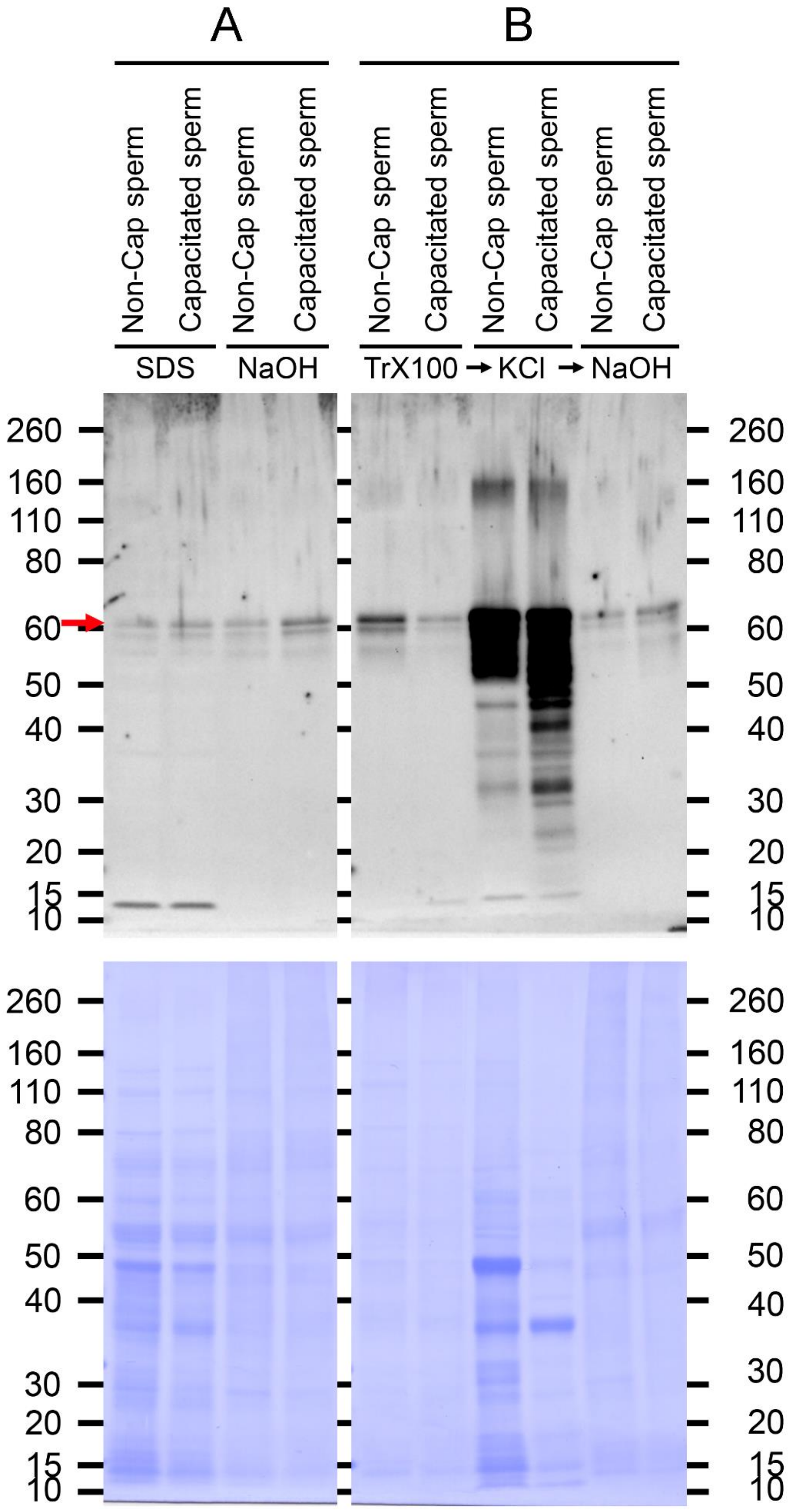
A
B
Non-Cap sperm
Capacitated sperm
Non-Cap sperm
Capacitated sperm
Non-Cap sperm
Capacitated sperm
Non-Cap sperm
Capacitated sperm
Non-Cap sperm
Capacitated sperm
SDS
NaOH
TrX100 → KCl → NaOH
260
160
110
80
60
50
40
30
20
15
10

Fig. S12.

Parallel (A) and sequential (B) isolation of perinuclear theca proteins and CCIN WB detection. Membranes were stained with the CBB after the chemiluminescence detection for protein normalization purposes. The corresponding CBB stained membrane is depicted under its WB detection complement. For the sequential isolation (B), the KCl fraction was precipitated and the whole precipitate (an equivalent of ~100 mil spermatozoa) was loaded on the gel; as opposed to 20 mil sperm equivalent in TrX-100 and NaOH fractions. It is apparent from the CBB stained membrane that the total protein load is the highest in the KCl fraction.

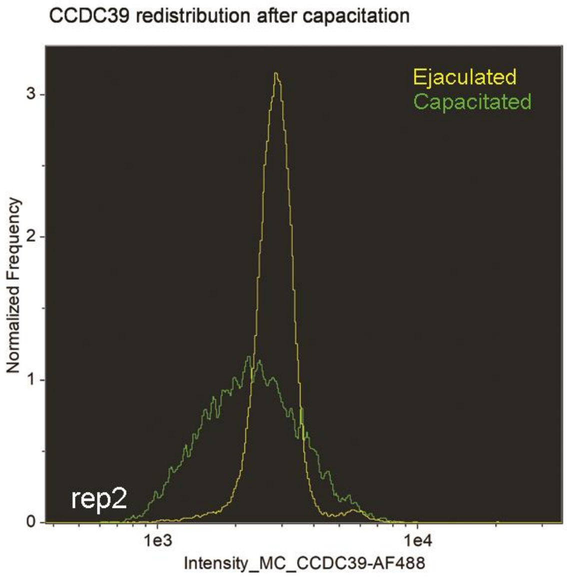

Intensity_MC_CCDC39-AF488

Population	Count	Objects/mL	Mean	Median	Std. Dev.	Mode
Capacitated Spermatozoa	6813	1057084.56	2498.59	2281.88	1103.51	2208.62
Non-Capac Spermatozoa	16776	2602913.63	2903.14	2839.67	1050.92	2936.74

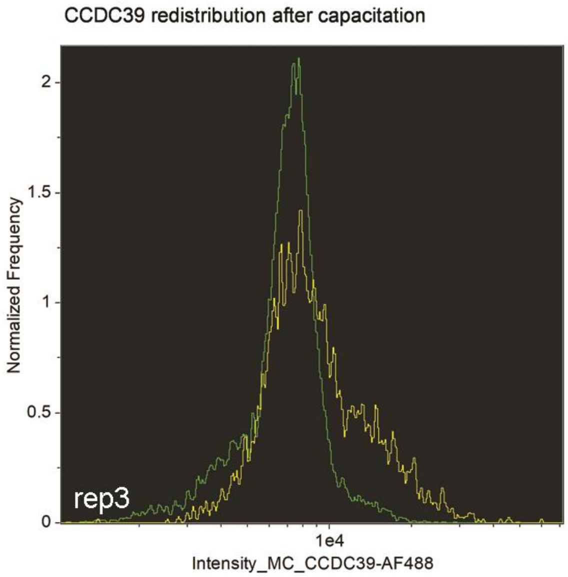

Intensity_MC_CCDC39-AF488

Population	Count	Objects/mL	Mean	Median	Std. Dev.	Mode
Non-Capac Spermatozoa	2726	1036949.78	9859.56	8326.38	5023.21	6938.45
Capacitated Spermatozoa	6547	2490429.27	7094.36	7094.75	2389.99	7569.92

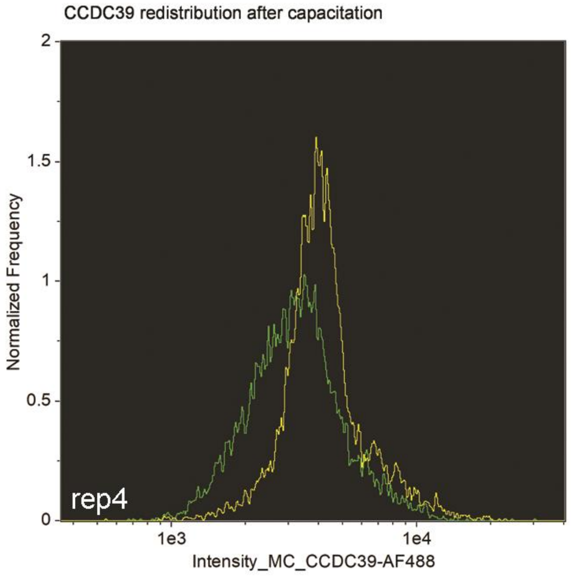

Intensity_MC_CCDC39-AF488

Population	Count	Objects/mL	Mean	Median	Std. Dev.	Mode
Non-Capac Spermatozoa	4691	742947.57	4536.17	4048.07	2099.83	4015.35
Capacitated Spermatozoa	4674	740255.16	3559.93	3208.54	1831.67	2808.68

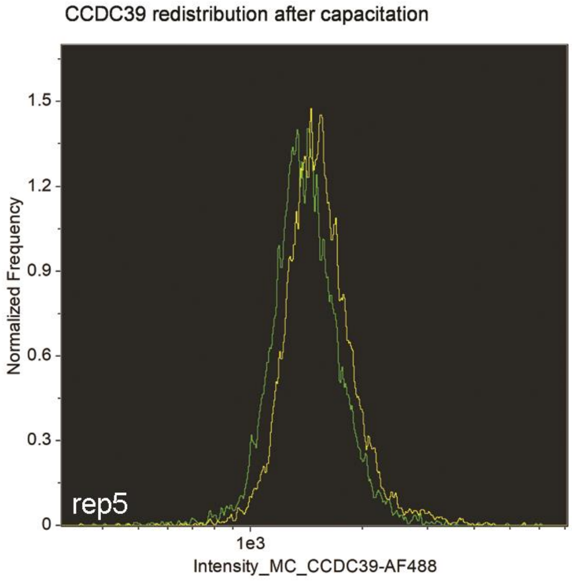

Intensity_MC_CCDC39-AF488

Population	Count	Objects/mL	Mean	Median	Std. Dev.	Mode
Non-Capac Spermatozoa	8072	4862893.2	1579.28	1513.19	449.07	1494.24
Capacitated Spermatozoa	6419	3867060.39	1446.91	1399.8	356.46	1316.83

Fig. S13.

The other four replicates of the capacitation-related CCDC39 redistribution from Fig 6G with the corresponding descriptive statistics.

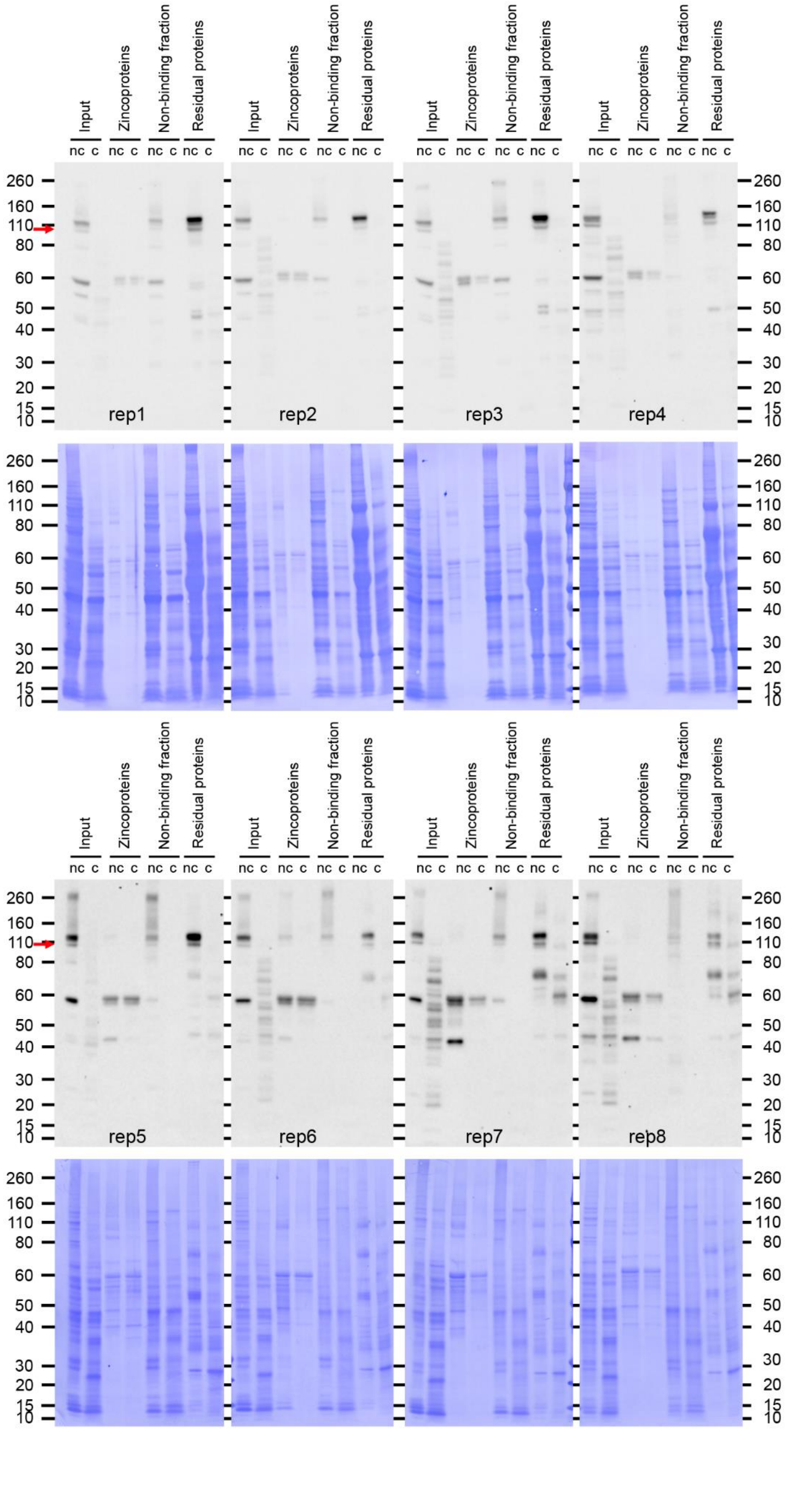
Input
Zincoproteins
Non-binding fraction
Residual proteins
nc c nc c nc c nc c
260
160
110
80
60
50
40
30
20
15
10
rep1
rep2
rep3
rep4
rep5
rep6
rep7
rep8

Fig. S14.

All eight replicates of CCDC39 WB detection from Fig 6H. Membranes were stained with the CBB after the chemiluminescence detection for protein normalization purposes. The corresponding CBB stained membrane is depicted under its WB detection complement.

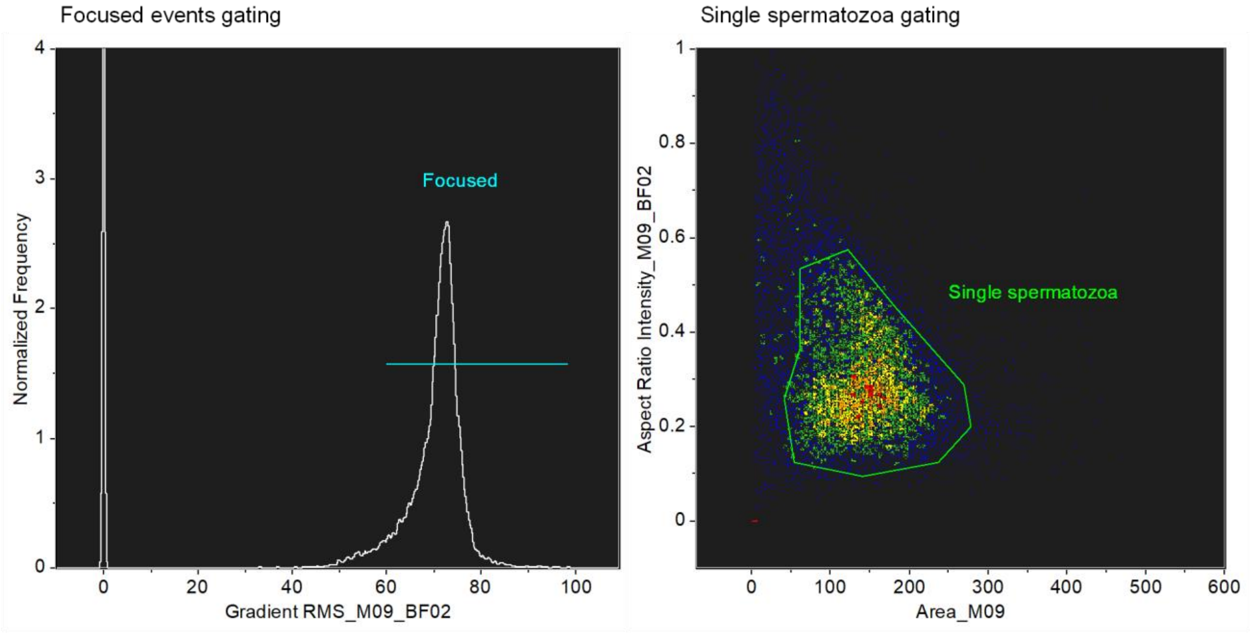

Gradient RMS_M09_BF02

Population	Count	%Gated
All	19141	100
Focused	15986	83.5

Area_M09

Population	Count	%Gated
Focused	15986	100
Single spermatozoa & Focused	12464	78

Fig. S15.

Gating approach - initial events collected from FlowSight data acquisition were gated for cells in focus as a function of brightfield gradient RMS, (a calculation of image crispness). Events in focus were further gated to analyze only single spermatozoa, plotted as brightfield intensity area by aspect ratio.

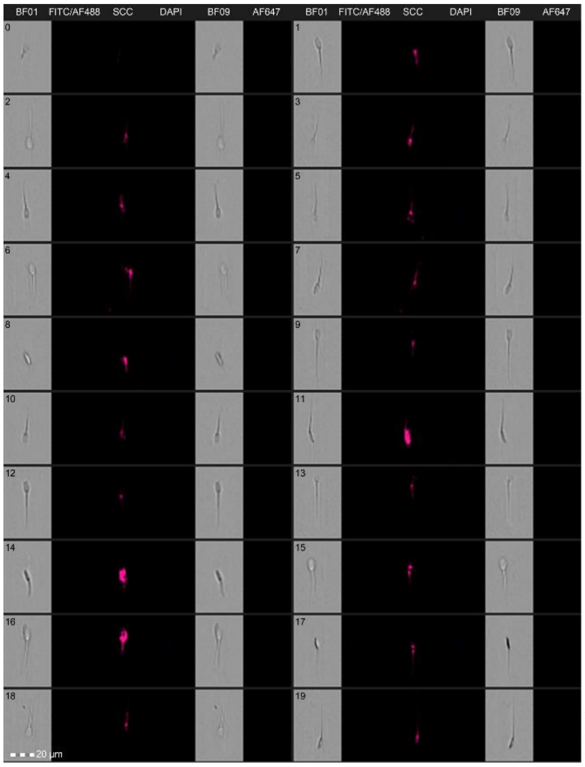

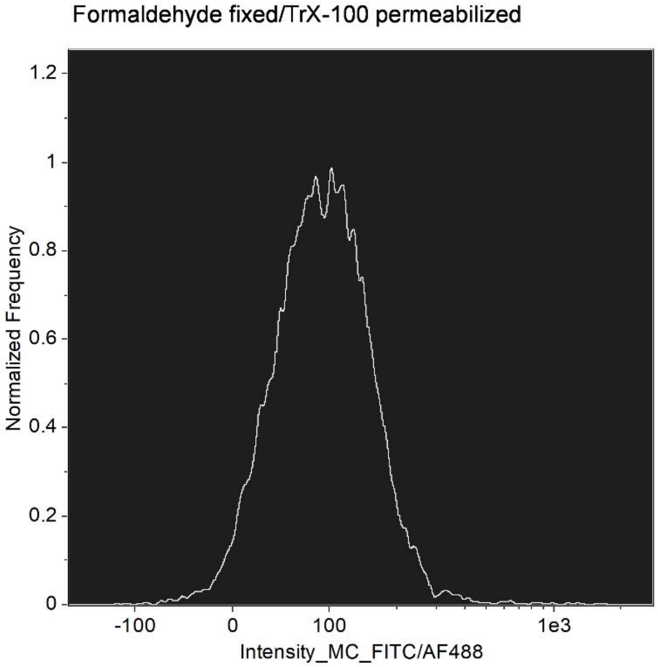

Intensity_MC_FITC/AF488

Population	Count	%Gated	Mean	Median	Std. Dev.	Mode	Variance
All	10000	100	99.6	92.67	75.44	95.89	5690.59

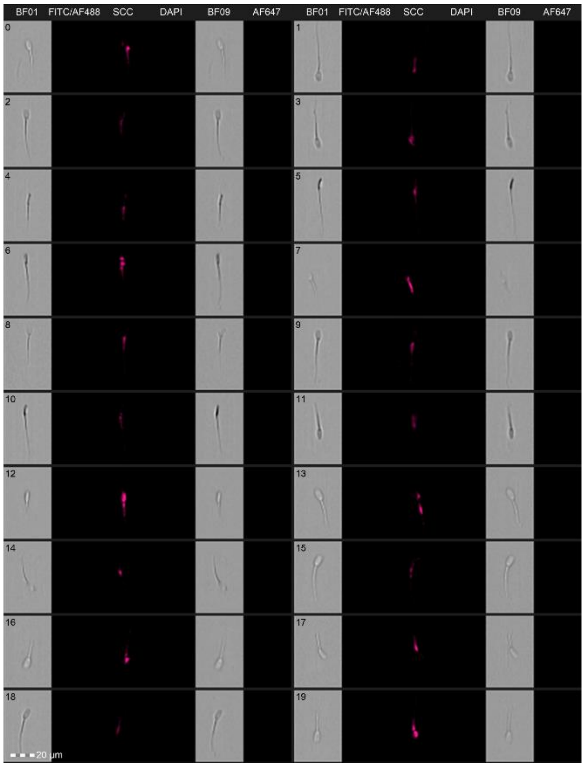

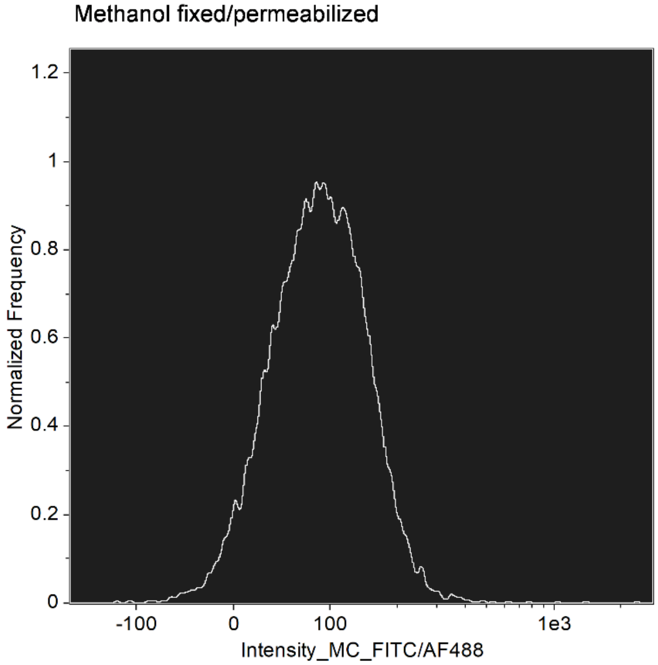

Intensity_MC_FITC/AF488

Population	Count	%Gated	Mean	Median	Std. Dev.	Mode	Variance
All	10000	100	92.48	88.81	64.19	90.35	4120.95

Fig. S16.

Secondary immunofluorescence controls. Formaldehyde fixed and Triton X-100 permeabilized spermatozoa were incubated with anti-CA2 or anti-CCIN primary antibodies, and methanol fixed/permeabilized spermatozoa were incubated with anti-CCDC39 primary antibody, respectively. Secondary incubation was void of secondary goat-anti-rabbit-FITC, DAPI, and PNA-

AF647. Image-based flowcytometry was run with the same laser/optics settings as the respective experiments.

Supplementary References

1 Mishra, A. K., Kumar, A., Swain, D. K., Yadav, S. & Nigam, R. Insights into pH regulatory mechanisms in mediating spermatozoa functions. *Veterinary world* **11**, 852-858, doi:10.14202/vetworld.2018.852-858 (2018).

2 Supuran, C. T. Carbonic anhydrases: novel therapeutic applications for inhibitors and activators. *Nature reviews. Drug discovery* **7**, 168-181, doi:10.1038/nrd2467 (2008).

3 José, O. *et al.* Carbonic anhydrases and their functional differences in human and mouse sperm physiology. *Biochemical and biophysical research communications* **468**, 713-718, doi:10.1016/j.bbrc.2015.11.021 (2015).

4 Wandernoth, P. M. *et al.* Normal Fertility Requires the Expression of Carbonic Anhydrases II and IV in Sperm. *The Journal of biological chemistry* **290**, 29202-29216, doi:10.1074/jbc.M115.698597 (2015).

5 Parkkila, S., Kaunisto, K., Kellokumpu, S. & Rajaniemi, H. A high activity carbonic anhydrase isoenzyme (CA II) is present in mammalian spermatozoa. *Histochemistry* **95**, 477-482, doi:10.1007/bf00315743 (1991).

6 Di Fiore, A., Supuran, C. T., Scaloni, A. & De Simone, G. Human carbonic anhydrases and post-translational modifications: a hidden world possibly affecting protein properties and functions. *Journal of enzyme inhibition and medicinal chemistry* **35**, 1450-1461, doi:10.1080/14756366.2020.1781846 (2020).

7 Longo, F. J., Krohne, G. & Franke, W. W. Basic proteins of the perinuclear theca of mammalian spermatozoa and spermatids: a novel class of cytoskeletal elements. *J Cell Biol* **105**, 1105-1120, doi:10.1083/jcb.105.3.1105 (1987).

8 Oko, R. & Maravei, D. Protein composition of the perinuclear theca of bull spermatozoa. *Biology of reproduction* **50**, 1000-1014, doi:10.1095/biolreprod50.5.1000 (1994).

9 Oko, R. & Maravei, D. Distribution and possible role of perinuclear theca proteins during bovine spermiogenesis. *Microscopy research and technique* **32**, 520-532, doi:10.1002/jemt.1070320605 (1995).

10 Lécuyer, C. *et al.* Actin-binding properties and colocalization with actin during spermiogenesis of mammalian sperm calicin. *Biology of reproduction* **63**, 1801-1810, doi:10.1095/biolreprod63.6.1801 (2000).

11 Oko, R., Aul, R., Wu, A. & Sutovsky, P. The sperm head cytoskeleton. *Andrology in the 21st Century. Medimond Publishing Company, Englewood*, 37-51 (2001).

12 Oko, R. & Sutovsky, P. Biogenesis of sperm perinuclear theca and its role in sperm functional competence and fertilization. *Journal of reproductive immunology* **83**, 2-7, doi:10.1016/j.jri.2009.05.008 (2009).

13 von Bülow, M., Heid, H., Hess, H. & Franke, W. W. Molecular nature of calicin, a major basic protein of the mammalian sperm head cytoskeleton. *Exp Cell Res* **219**, 407-413, doi:10.1006/excr.1995.1246 (1995).

14 Kerns, K., Zigo, M., Drobnis, E. Z., Sutovsky, M. & Sutovsky, P. Zinc ion flux during mammalian sperm capacitation. *Nature communications* **9**, 2061, doi:10.1038/s41467-018-04523-y (2018).

15 Kerns, K. *et al.* Sperm Cohort-Specific Zinc Signature Acquisition and Capacitation-Induced Zinc Flux Regulate Sperm-Oviduct and Sperm-Zona Pellucida Interactions. *International journal of molecular sciences* **21**, doi:10.3390/ijms21062121 (2020).

16 Blanchon, S. *et al.* Delineation of CCDC39/CCDC40 mutation spectrum and associated phenotypes in primary ciliary dyskinesia. *J Med Genet* **49**, 410-416, doi:10.1136/jmedgenet-2012-100867 (2012).
17 Merveille, A. C. *et al.* CCDC39 is required for assembly of inner dynein arms and the dynein regulatory complex and for normal ciliary motility in humans and dogs. *Nat Genet* **43**, 72-78, doi:10.1038/ng.726 (2011).
18 Sironen, A., Shoemark, A., Patel, M., Loebinger, M. R. & Mitchison, H. M. Sperm defects in primary ciliary dyskinesia and related causes of male infertility. *Cellular and molecular life sciences : CMLS* **77**, 2029-2048, doi:10.1007/s00018-019-03389-7 (2020).
