# Supplementary material for: Zinc is a master-regulator of sperm function associated with binding, motility, and metabolic modulation during porcine sperm capacitation
Source: Commun Biol. 2022 Jun 3;5:538. doi: 10.1038/s42003-022-03485-8 (PMC9166710; doi:10.1038/s42003-022-03485-8)
Supplement: Supplementary file 3 — Description of Additional Supplementary Files [file 42003_2022_3485_MOESM3_ESM.pdf]

Description of Additional Supplementary Files

File name: Supplementary Data 1
Description: List of identified UniProtKB accessions, spectral counts, and normalization.

File name: Supplementary Data 2
Description: Panther analysis data, volcano plot data.

File name: Supplementary Data 3
Description: Summary of identified zincoproteins significantly different in abundance between ejaculated and *in vitro* capacitated spermatozoa; including UniProt.org entries, theoretical molecular mass, function, and localization in spermatozoa based on corresponding references.

File name: Supplementary Data 4
Description: Pie charts data.

File name: Supplementary Data 5
Description: Prediction of zinc-binding sites data.
