# Supplementary material for: Zinc is a master-regulator of sperm function associated with binding, motility, and metabolic modulation during porcine sperm capacitation
Source: Commun Biol. 2022 Jun 3;5:538. doi: 10.1038/s42003-022-03485-8 (PMC9166710; doi:10.1038/s42003-022-03485-8)
Supplement: Supplementary file 8 — Supplementary Data 5 [file 42003_2022_3485_MOESM8_ESM.docx]

| **HUGO names** | **Protein names** | **uniprot.org Entry** | **Avg. Mass** | **Molecular function in spermatozoa** | **Localization in spermatozoa** | **Reference** |
| --- | --- | --- | --- | --- | --- | --- |
| ABHD10 | Abhydrolase domain containing 10 | A0A287AL53 | 33,825 | Redox homeostasis (assumed in spermatozoa) | Midpiece, mitochondria | ^1^ |
| ACADSB | Acyl-CoA dehydrogenase short/branched chain | F1SED0 | 40,690 | Energy metabolism, fatty acid beta oxidation | Midpiece, mitochondria | ^2,3^ |
|  |  | A0A287B1V1 | 47,260 |  |  |  |
| ACADVL | Acyl-CoA dehydrogenase very long chain | F1ST43 | 70,327 |  |  |  |
|  |  | A0A0B8RTA8 |  |  |  |  |
| ACTN1 | Actinin alpha 1 | I3LLY3 | 102,322 | Cytoskeleton, actin filament bundle assembly, and filament network formation | Sperm head, principal piece | ^4,5^ |
| ACTN4 | Actinin alpha 4 | A0A0B8S0C5 | 104,905 |  |  |  |
|  |  | A0A287A4Q7 |  |  |  |  |
|  |  | A0A287AA30 | 117,026 |  |  |  |
| AK7 | Adenylate kinase 7 isoform 1 | F1SAQ8 | 82,887 | Energy metabolism, cellular energy homeostasis | Flagellum | ^6,7^ |
| ALDOA | Fructose-bisphosphate aldolase (EC 4.1.2.13) | A0A287B8F3 | 44,999 | Energy metabolism, glycolysis | Sperm head, acrosomal region, principal piece | ^8,9^ |
| ALMS1 | ALMS1 centrosome and basal body associated protein | A0A286ZKT4 | 474,832 | Sperm motility, sperm elongation | Flagellum, basal body of flagellum, proximal centriole | ^10,11^ |
|  |  | A0A287AH92 | 481,619 |  |  |  |
| AQN1 | Spermadhesin AQN1 | P26322 | 11,882 | Fertilization, zona pellucida binding, formation of the oviductal epithelium reservoir | Sperm head, acrosomal region, and flagellum | ^12-21^ |
|  |  | Q4R0H3 | 13,989 |  |  |  |
| ARMC12 | Armadillo repeat containing 12 | F1RYX9 | 38,747 | Spermatogenesis | Not known in spermatozoa | ^22^ |
| ATP5C1/  ATP5F1C | ATP synthase F1 subunit gamma | A0A0B8RW24 | 32,992 | Energy metabolism, oxidative phosphorylation | Midpiece, mitochondria | ^23^ |
|  |  | A0A287A9I8 |  |  |  |  |
|  |  | A0A287AHM1 | 32,863 |  |  |  |
| AWN | Spermadhesin AWN | Q4R0H8 | 16,902 | Fertilization, zona pellucida binding | Sperm head, acrosomal region, and flagellum | ^13,16,19,24-26^ |
| BAG6 | BCL2-associated athanogene 6 | A0A2C9F3H8 | 118,540 | Fertilization, HSPA2-stabilizing protein | Sper head, equatorial region in ejaculated sperm, and acrosomal region in capacitated sperm | ^27^ |
|  |  | A5D9M6 | 118,627 |  |  |  |
|  |  | A0A2C9F389 | 122,189 |  |  |  |
|  |  | A0A068C697 | 122,276 |  |  |  |
| BLVRB | Biliverdin reductase B (EC 1.3.1.24) | I3LQH7 | 22,213 | Redox homeostasis (assumed) | Sperm head, post equatorial region | ^28^ |
| C1orf56/ C4H1orf56 | Chromosome 1 open reading frame 56 / methylated in normal thymocytes / MENT | F1SSA1 | 37,745 | Not known in spermatozoa (proto-oncogene in somatic cells) | Not known in spermatozoa | ^29,30^ |
| CA2 | Carbonic anhydrase 2 isoform 1 | F1RXC2 | 29,258 | pH homeostasis | Flagellum, Principal piece | ^31^ |
| CABYR | Calcium binding tyrosine-(Y)-phosphorylation regulated transcript variant 3 | I6R469 | 41,275 | Signal transduction pathway | Flagellum, Principal piece | ^32^ |
| CCDC39 | Coiled-coil domain containing 39 | F1SGC2 | 110,108 | Sperm motility, assembly of dynein regulatory complex and inner dynein arm complexes | Flagellum | ^33,34^ |
| CCDC40 | Coiled-coil domain containing 40 | F1RZ80 | 110,615 | Sperm motility, assembly of dynein regulatory complex and inner dynein arm complexes | Flagellum | ^35^ |
|  |  | I3LAH7 | 113,389 |  |  |  |
| CCDC42 | Coiled-coil domain-containing 42 | F1SSA4 | 37,828 | Sperm motility, assembly of dynein regulatory complex and inner dynein arm complexes | Flagellum | ^36^ |
| CCDC136 | Coiled-coil domain containing 136 | F1SMN3 | 133,275 | Fertilization | Sperm head, acrosomal region | ^37^ |
|  |  | F1SMN4 | 133,510 |  |  |  |
| CCDC196 | Coiled-coil domain containing 196 | F1SA53 | 28,448 | Not known in spermatozoa | Not known in spermatozoa |  |
| CCIN | Calicin | F1ST87 | 66,818 | Cytoskeleton | Sperm head, perinuclear theca | ^38-41^ |
| CFAP57 | Cilia-and flagella-associated protein 57 | I3LHD4 | 130,346 | Sperm motility (assumed on related CFAP43, CFAP44 and CFAP69) | Flagellum (assumed) | ^42-44^ |
| CFAP58 | Cilia-and flagella-associated protein 58 | F1S5N5 | 103,711 |  |  |  |
| CISD1 | CDGSH iron-sulfur domain-containing protein 1 | X5FUA3 | 11,965 | Normal function of mitochondria | Midpiece, mitochondria | ^45^ |
| CLMN | Calmin | F1SAR7 | 111,102 | Not known in spermatozoa | Not known in spermatozoa, expressed in later stages of maturing spermatogenic cells | ^46^ |
| CNDP2 | Carnosine dipeptidase 2 (cytosolic nonspecific dipeptidase) | F1SNL7 | 52,734 | Not known in spermatozoa | Not known in spermatozoa, present in seminal plasma | ^47-49^ |
| DEFB112 | Defensin beta 112 | A0A287BBL9 | 15,735 | Regulation of infection, control of spermatozoa maturation | Not known in spermatozoa, expressed in epididymis | ^50^ |
| DHRS11 | Dehydrogenase/reductase 11 | F1S1B9 | 35,296 | Steroid biosynthetic process | Not known in spermatozoa, expressed in testes, small intestine, colon, kidney | ^51^ |
| DLAT | Dihydrolipoamide S-acetyltransferase (Acetyltransferase component of pyruvate dehydrogenase complex) | F1SMB2 | 69,184 | Energy metabolism, oxidative phosphorylation | Midpiece, mitochondria | ^52^ |
| DNAH7 | Dynein axonemal heavy chain 7 | A0A287AHL5 | 460,563 | Sperm motility | Flagellum | ^53-55^ |
| DNAH12 | Dynein axonemal heavy chain 12 | I3LP90 | 365,587 |  |  |  |
| DNAH17 | Dynein axonemal heavy chain 17 | A0A287AFU3 | 502,796 |  |  |  |
| DNAI4/ WDR78 | Dynein axonemal intermediate chain 4 | I3LB78 | 90,936 |  |  |  |
| DRC3/ LRRC48 | Dynein regulatory complex subunit 3 | A0A287ABI6 | 58,676 | Sperm motility, assembly of dynein regulatory complex and inner dynein arm complexes | Flagellum | ^56,57^ |
| EFHC1 | EF-hand domain containing 1 | F1S7E5 | 74,016 | Sperm motility | Flagellum | ^58,59^ |
| ENKUR | Enkurin, TRPC channel interacting protein | A0A286ZIE6 | 29,891 | Fertilization, acrosomal exocytosis, signal transduction pathway | Sperm head, acrosomal region | ^60^ |
| ETFA | Electron transfer flavoprotein subunit alpha | A0A286ZRJ3 | 36,430 | Energy metabolism, fatty acid beta oxidation, oxidative phosphorylation | Midpiece, mitochondria | ^61^ |
|  |  | A0A287A4T2 | 35,052 |  |  |  |
|  |  | F1SJX1 | 32,768 |  |  |  |
|  |  | Q6UAQ9 | 32,884 |  |  |  |
| ETFB | Electron transfer flavoprotein subunit beta | Q6UAQ8 | 27,764 |  |  |  |
| FAM71B | Family with sequence similarity 71 member B | F1RQD7 | 61,392 | Not known in spermatozoa | Sperm head, nucleus | ^62^ |
| FAM166A | Family with sequence similarity 166 member A, HSD46 | F1RVX7 | 36,265 | Not known in spermatozoa | Not known in spermatozoa | ^63,64^ |
| FAM205C | DUF4599 domain-containing protein | A0A287AEH4 | 42,478 | Not known in spermatozoa | Not known in spermatozoa |  |
| FCGBP | Fc fragment of IgG binding protein | A0A287BCE6 | 265,392 | Reduction of the anti-sperm antibodies binding | Not known in spermatozoa | ^65-67^ |
| GK2 | Glycerol kinase 2 | F1RVC1 | 60,651 | Sperm motility, proper functioning of mitochondria | Midpiece, mitochondria | ^68^ |
| GPHN | Gephyrin [Molybdopterin molybdenumtransferase (EC 2.10.1.1)] | A0A286ZKW0 | 85,018 | Not known in spermatozoa | Not known in spermatozoa |  |
|  |  | A0A287AFJ9 | 87,084 |  |  |  |
|  |  | A0A287BE36 | 86,487 |  |  |  |
|  |  | A0A287B952 | 83,567 |  |  |  |
|  |  | F1SA44 | 79,748 |  |  |  |
| HADHB | Hydroxyacyl-CoA dehydrogenase trifunctional multienzyme complex subunit beta | F1SDN2 | 51,250 | Energy metabolism, fatty acid beta oxidation | Midpiece, mitochondria | ^69^ |
| HIP1 | Huntingtin interacting protein 1 | F1RKC0 | 110,894 | Cytoskeleton, stabilizing actin, and microtubules | Sperm head, cytosol | ^70,71^ |
|  |  | A0A0B8RZH9 | 113,499 |  |  |  |
| HSPA9 | Heat shock protein family A (Hsp70) member 9 | A0A287ADJ2 | 73,651 | Molecular chaperone, protein refolding | Sperm head and midpiece | ^72-74^ |
| HSPD1 | Heat shock protein family D (Hsp60) member 1 | F1SMZ7 | 60,907 | Molecular chaperone, protein refolding | Midpiece, mitochondria | ^74^ |
| HYDIN | HYDIN axonemal central pair apparatus protein | A0A286ZP34 | 565,224 | Sperm motility | Flagellum | ^75^ |
| IGBP1 | Immunoglobulin binding protein 1 | K7GQ35 | 38,993 | Signal transduction pathway, regulation | Not known in spermatozoa | ^76^ |
| IMMT | Inner membrane mitochondrial protein/Mic60/mitofilin | A0A287AMM6 | 82,671 | Normal function of mitochondria | Midpiece, mitochondria | ^77^ |
| IQCN | IQ motif containing N | A0A287AI93 | 82,674 | Signal transduction pathway | Not known in spermatozoa | ^78^ |
| ISYNA1 | Inositol-3-phosphate synthase 1 | A0A287AXJ7 | 60,785 | Sperm motility, sperm maturation, osmolarity regulation (assumed) | Not known in spermatozoa | ^79^ |
|  |  | A0A0B8RVT0 |  |  |  |  |
| LDHAL6B | L-lactate dehydrogenase (EC 1.1.1.27) | A0A0K0KW08 | 41,861 | Energy metabolism, conversion of lactate to pyruvate | Sperm head, and midpiece | ^80^ |
| LRRC37A3 | Leucine rich repeat containing 37 member A3 | F1RRQ6 | 94,348 | Not known in spermatozoa | Not known in spermatozoa |  |
| LRRC37B | Leucine rich repeat containing 37B | I3L8Z8 | 96,079 | Sperm motility | Flagellum (assumed) | ^81,82^ |
| MCCC1 | Methylcrotonoyl-CoA carboxylase 1 (EC 6.4.1.4) | A0A287A8I4 | 77,214 | Energy metabolism, leucine metabolism | Midpiece, mitochondria | ^83,84^ |
| MDH1 | Malate dehydrogenase 1 | P11708 | 36,454 | Energy metabolism, oxidative phosphorylation | Midpiece, mitochondria | ^85^ |
| MMAB | Corrinoid adenosyltransferase (EC 2.5.1.17) / ATP:cob(I)alamin adenosyltransferase / cilia and flagella associated protein 23 | F1RIS6 | 26,535 | Not known in spermatozoa | Not known in spermatozoa | ^86^ |
| NME2 | Nucleoside diphosphate kinase B (EC 2.7.4.6) | Q2EN76 | 17,176 | Signal transduction pathway (assumed) | Flagellum | ^87,88^ |
| NUP54 | Nucleoporin 54 | A0A0B8RVN3 | 55,996 | Nuclear pores | Sperm head, nucleus | ^89^ |
|  |  | A0A286ZTU0 |  |  |  |  |
|  |  | F1RYT5 | 61,258 |  |  |  |
| NUP58/ NUPL1 | Nucleoporin 58 | A0A287B8D6 | 56,838 | Nuclear pores | Sperm head, nucleus |  |
|  |  | M3TYS2 | 59,196 |  |  |  |
| ODF2 | Outer dense fiber of sperm tails 2 | F1RR82 | 99,196 | Sperm motility | Flagellum | ^90,91^ |
| PACRG | Parkin coregulated (Parkin coregulated gene protein isoform 2) | A0A287AL41 | 29,373 | Normal function of mitochondria | Midpiece, mitochondria | ^92,93^ |
| PCMT1 | Protein-L-isoaspartate (D-aspartate) O-methyltransferase (EC 2.1.1.77) | J9JIK8 | 30,294 | Repair of age-damaged proteins (assumed) | Not known in spermatozoa | ^94,95^ |
|  |  | P80895 | 24,646 |  |  |  |
| PDHA1 | Pyruvate dehydrogenase E1 component subunit alpha 1 (EC 1.2.4.1) | I3LCI2 | 43,365 | Energy metabolism, oxidative phosphorylation, regulation of capacitation via the modulation of sperm intracellular lactate, pH, and calcium | Midpiece, mitochondria | ^52,96^ |
| PDHB | Pyruvate dehydrogenase E1 component subunit beta (EC 1.2.4.1) | F1SGH5 | 39,273 |  |  |  |
| PDZD9 | PDZ domain containing 9 | I3LN27 | 29,393 | Not known in spermatozoa | Not known in spermatozoa |  |
| PEBP4 | Phosphatidylethanolamine-binding protein 4 | C4TP28 | 25,177 | Sperm motility | Not known in spermatozoa | ^97^ |
| PLCZ1 | Phosphoinositide phospholipase C zeta 1 (EC 3.1.4.11) | F1SQZ0 | 73,754 | Signal transduction pathway, sperm borne oocyte activating factor | Sperm head, acrosomal region | ^98-100^ |
| PPEF1 | Protein Phosphatase With EF-Hand Domain 1 (PP1, EC 3.1.3.16) | K7GM53 | 69,552 | Sperm motility, epididymal maturation | Sperm head, and flagellum | ^101,102^ |
|  |  | I3LR47 | 75,253 |  |  |  |
| PPM1B | PPM-type phosphatase domain-containing protein (Ser/Thr phosphatase 2Cβ) | F1S5K0 | 52,388 | Signal transduction pathway | Midpiece, mitochondria | ^103^ |
| PRUNE1 | Prune exopolyphosphatase 1 (EC 3.6.1.11) | A0A0B8RZ88 | 49,947 | Not known in spermatozoa | Not known in spermatozoa |  |
|  |  | F1SS99 |  |  |  |  |
| PSMA3 | Proteasome subunit alpha type (EC 3.4.25.1) | F1SSL6 | 28,433 | Proteasome-mediated protein degradation | Sperm head, acrosomal region, connecting piece | ^104-107^ |
| PSMA8 | Proteasome subunit alpha type (EC 3.4.25.1) | F1SBA5 | 27,928 |  |  |  |
| PSMB1 | Proteasome subunit beta (EC 3.4.25.1) | A0A287BPP9 | 26,330 |  |  |  |
| PSMB4 | Proteasome subunit beta (EC 3.4.25.1) | A0A287B088 | 29,053 |  |  |  |
| PTPA | Serine/threonine-protein phosphatase 2A activator (EC 5.2.1.8) | A0A286ZTF3 | 36,834 | Regulation of protein phosphatase 2 activity | Not known in spermatozoa | ^108^ |
|  |  | A0A287ARC8 | 41,385 |  |  |  |
| RAB2B | Ras-related protein Rab-2B isoform 1 | F1S8J6 | 24,236 | Fertilization, acrosomal exocytosis (assumed) | Sperm head, acrosomal region | ^109-111^ |
| RGS22 | Regulator of G protein signaling 22 | F1S0P1 | 146,170 | Signal transduction pathway | Sperm head, principal piece | ^112^ |
| RNASE9 | Ribonuclease A family member 9 (inactive) | A0A287AUN3 | 25,293 | Regulation of infection, control of spermatozoa maturation | Sperm head | ^113,114^ |
| S100A14 | S100 calcium binding protein A14 | F1SFV3 | 11,444 | Not known in spermatozoa | Not known in spermatozoa | ^115^ |
| SAXO1 | Stabilizer of axonemal microtubules 1 | A0A286ZNM0 | 52,513 | Sperm motility | Flagellum, proximal centriole | ^116^ |
| SCCPDH | Saccharopine dehydrogenase (putative) | F1S8P1 | 47,333 | Energy metabolism, lysine metabolism | Not known in spermatozoa | ^117^ |
| SPATA19 | Spermatogenesis-associated protein 19, mitochondrial | I3LDJ2 | 17,970 | Sperm motility, proper mitochondrial function | Midpiece, mitochondria | ^118^ |
| SPEM3 | SPEM family member 3 | A0A287AQA6 | 125,423 | Not known in spermatozoa | Not known in spermatozoa |  |
| SPR | Short-Chain Dehydrogenase/Reductase Family Protein 38C, member 1 (SDR38C1), related to DCXR/P34H/SDR20C1 | A0A286ZKH3 | 28,774 | Fertilization, zona pellucida binding (assumed) | Sperm head, acrosomal region (assumed) | ^119-127^ |
| SQOR | Sulfide:quinone oxidoreductase, mitochondrial (SQRDL) | F1SN58 | 50,125 | Production of H_2_S | Midpiece, mitochondria | ^128^ |
| STKLD1 | Serine/threonine kinase like domain containing 1 (SGK71) | A0A287ANN3 | 71,921 | Signal transduction pathway (assumed) | Not known in spermatozoa | ^129^ |
| SUGT1 | SGT1 homolog, MIS12 kinetochore complex assembly cochaperone | A0A286ZQC1 | 38,290 | Not known in spermatozoa | Not known in spermatozoa | ^130^ |
| TEKT3 | Tektin 3 | F1SDE8 | 56,473 | Sperm motility | Sperm head, flagellum | ^131-134^ |
| TEX33 | Testis expressed 33 | F1SKL3 | 30,197 | Not known in spermatozoa | Sperm head, flagellum | ^135,136^ |
| TIPRL | TOR signaling pathway regulator | A0A287BCR7 | 27,486 | Signal transduction pathway, regulation of protein phosphatase 2A | Sperm head, principal piece | ^137^ |
|  |  | F1RPV1 | 31,425 |  |  |  |
| TSTD1 | Thiosulfate sulfurtransferase like domain containing 1 | F1S179 | 14,953 | Production of H_2_S | Not known in spermatozoa | ^138^ |
| TUBB4B | Tubulin beta 4B | A0A287A275 | 49,831 | Cytoskeleton | Sperm head, flagellum | ^139,140^ |
| VAPA | VAMP associated protein A | A0A287BBB5 | 32,235 | Not known in spermatozoa | Not known in spermatozoa |  |
| VAPB | VAMP associated protein B | A0A287B186 | 27,037 |  |  |  |
|  |  | A5GFS8 | 27,053 |  |  |  |
|  | Uncharacterized protein (EF-hand superfamily) | F1SJU0 | 31,603 | Signal transduction pathway (assumed) | Not known in spermatozoa |  |
|  | Uncharacterized protein (protein phosphatase 2 inhibitor family) | F1SFT7 | 54,899 | Signal transduction pathway, regulation of phosphoprotein phosphatase activity (assumed) | Not known in spermatozoa |  |

**Supplementary References**

1 Cao, Y. *et al.* ABHD10 is an S-depalmitoylase affecting redox homeostasis through peroxiredoxin-5. *Nat Chem Biol* **15**, 1232-1240, doi:10.1038/s41589-019-0399-y (2019).

2 De Amicis, F. *et al.* Progesterone through progesterone receptors affects survival and metabolism of pig sperm. *Anim Reprod Sci* **135**, 75-84, doi:10.1016/j.anireprosci.2012.09.004 (2012).

3 Santoro, M. *et al.* Sperm metabolism in pigs: a role for peroxisome proliferator-activated receptor gamma (PPARγ). *J Exp Biol* **216**, 1085-1092, doi:10.1242/jeb.079327 (2013).

4 Yagi, A. & Paranko, J. Localization of actin, alpha-actinin, and tropomyosin in bovine spermatozoa and epididymal epithelium. *Anat Rec* **233**, 61-74, doi:10.1002/ar.1092330109 (1992).

5 Yagi, A. & Paranko, J. Actin, alpha-actinin, and spectrin with specific associations with the postacrosomal and acrosomal domains of bovine spermatozoa. *Anat Rec* **241**, 77-87, doi:10.1002/ar.1092410111 (1995).

6 Fernandez-Gonzalez, A., Kourembanas, S., Wyatt, T. A. & Mitsialis, S. A. Mutation of murine adenylate kinase 7 underlies a primary ciliary dyskinesia phenotype. *Am J Respir Cell Mol Biol* **40**, 305-313, doi:10.1165/rcmb.2008-0102OC (2009).

7 Lorès, P. *et al.* Homozygous missense mutation L673P in adenylate kinase 7 (AK7) leads to primary male infertility and multiple morphological anomalies of the flagella but not to primary ciliary dyskinesia. *Human molecular genetics* **27**, 1196-1211, doi:10.1093/hmg/ddy034 (2018).

8 Chiquete-Felix, N. *et al.* In guinea pig sperm, aldolase A forms a complex with actin, WAS, and Arp2/3 that plays a role in actin polymerization. *Reproduction (Cambridge, England)* **137**, 669-678, doi:10.1530/rep-08-0353 (2009).

9 Krisfalusi, M., Miki, K., Magyar, P. L. & O'Brien, D. A. Multiple glycolytic enzymes are tightly bound to the fibrous sheath of mouse spermatozoa. *Biology of reproduction* **75**, 270-278, doi:10.1095/biolreprod.105.049684 (2006).

10 Arsov, T. *et al.* Fat aussie--a new Alström syndrome mouse showing a critical role for ALMS1 in obesity, diabetes, and spermatogenesis. *Mol Endocrinol* **20**, 1610-1622, doi:10.1210/me.2005-0494 (2006).

11 Collin, G. B. *et al.* Alms1-disrupted mice recapitulate human Alström syndrome. *Human molecular genetics* **14**, 2323-2333, doi:10.1093/hmg/ddi235 (2005).

12 Calvete, J. J., Carrera, E., Sanz, L. & Töpfer-Petersen, E. Boar spermadhesins AQN-1 and AQN-3: oligosaccharide and zona pellucida binding characteristics. *Biol Chem* **377**, 521-527, doi:10.1515/bchm3.1996.377.7-8.521 (1996).

13 Dostalova, Z., Calvete, J. J. & Topfer-Petersen, E. Interaction of non-aggregated boar AWN-1 and AQN-3 with phospholipid matrices. A model for coating of spermadhesins to the sperm surface. *Biological chemistry Hoppe-Seyler* **376**, 237-242 (1995).

14 Ekhlasi-Hundrieser, M. *et al.* Spermadhesin AQN1 is a candidate receptor molecule involved in the formation of the oviductal sperm reservoir in the pig. *Biology of reproduction* **73**, 536-545, doi:10.1095/biolreprod.105.040824 (2005).

15 Ensslin, M. *et al.* Identification by affinity chromatography of boar sperm membrane-associated proteins bound to immobilized porcine zona pellucida. Mapping of the phosphorylethanolamine-binding region of spermadhesin AWN. *Biological chemistry Hoppe-Seyler* **376**, 733-738 (1995).

16 Jonakova, V. *et al.* Spermadhesins of the AQN and AWN families, DQH sperm surface protein and HNK protein in the heparin-binding fraction of boar seminal plasma. *Journal of reproduction and fertility* **114**, 25-34, doi:10.1530/jrf.0.1140025 (1998).

17 Jonakova, V. *et al.* Isolation and biochemical characterization of a zona pellucida-binding glycoprotein of boar spermatozoa. *FEBS letters* **280**, 183-186 (1991).

18 Jonakova, V., Ticha, M., Kraus, M. & Cechova, D. Multifunctional sperm protein in gametic interaction. *Fertilität* **11**, 115-118 (1995).

19 Sanz, L., Calvete, J. J., Jonakova, V. & Topfer-Petersen, E. Boar spermadhesins AQN-1 and AWN are sperm-associated acrosin inhibitor acceptor proteins. *FEBS letters* **300**, 63-66 (1992).

20 Tichá, M., Kraus, M., Cechová, D. & Jonáková, V. Saccharide-binding properties of boar AQN spermadhesins and DQH sperm surface protein. *Folia biologica* **44**, 15-21 (1998).

21 Veselsky, L., Jonakova, V., Sanz, M. L., Topfer-Petersen, E. & Cechova, D. Binding of a 15 kDa glycoprotein from spermatozoa of boars to surface of zona pellucida and cumulus oophorus cells. *Journal of reproduction and fertility* **96**, 593-602 (1992).

22 Razavi, S. M. *et al.* Comprehensive functional enrichment analysis of male infertility. *Scientific Reports* **7**, 15778, doi:10.1038/s41598-017-16005-0 (2017).

23 Neupane, P., Bhuju, S., Thapa, N. & Bhattarai, H. K. ATP Synthase: Structure, Function and Inhibition. *Biomol Concepts* **10**, 1-10, doi:10.1515/bmc-2019-0001 (2019).

24 Dostalova, Z., Calvete, J. J., Sanz, L. & Topfer-Petersen, E. Quantitation of boar spermadhesins in accessory sex gland fluids and on the surface of epididymal, ejaculated and capacitated spermatozoa. *Biochimica et biophysica acta* **1200**, 48-54 (1994).

25 Petrunkina, A. M., Harrison, R. A. & Topfer-Petersen, E. Only low levels of spermadhesin AWN are detectable on the surface of live ejaculated boar spermatozoa. *Reproduction, fertility, and development* **12**, 361-371 (2000).

26 Rodriguez-Martinez, H., Iborra, A., Martinez, P. & Calvete, J. J. Immunoelectronmicroscopic imaging of spermadhesin AWN epitopes on boar spermatozoa bound in vivo to the zona pellucida. *Reproduction, fertility, and development* **10**, 491-497 (1998).

27 Bromfield, E., Aitken, R. J. & Nixon, B. Novel characterization of the HSPA2-stabilizing protein BAG6 in human spermatozoa. *Molecular human reproduction* **21**, 755-769, doi:10.1093/molehr/gav041 (2015).

28 D'Amours, O. *et al.* Evidences of Biological Functions of Biliverdin Reductase A in the Bovine Epididymis. *Journal of cellular physiology* **231**, 1077-1089, doi:10.1002/jcp.25200 (2016).

29 Alkebsi, L. *et al.* DNMT3B7 expression related to MENT expression and its promoter methylation in human lymphomas. *Leuk Res* **37**, 1662-1667, doi:10.1016/j.leukres.2013.09.014 (2013).

30 Hlady, R. A. *et al.* Loss of Dnmt3b function upregulates the tumor modifier Ment and accelerates mouse lymphomagenesis. *J Clin Invest* **122**, 163-177, doi:10.1172/jci57292 (2012).

31 Wandernoth, P. M. *et al.* Normal Fertility Requires the Expression of Carbonic Anhydrases II and IV in Sperm. *The Journal of biological chemistry* **290**, 29202-29216, doi:10.1074/jbc.M115.698597 (2015).

32 Kim, Y. H. *et al.* Translation and assembly of CABYR coding region B in fibrous sheath and restriction of calcium binding to coding region A. *Developmental biology* **286**, 46-56, doi:10.1016/j.ydbio.2005.07.005 (2005).

33 Merveille, A. C. *et al.* CCDC39 is required for assembly of inner dynein arms and the dynein regulatory complex and for normal ciliary motility in humans and dogs. *Nat Genet* **43**, 72-78, doi:10.1038/ng.726 (2011).

34 Sironen, A., Shoemark, A., Patel, M., Loebinger, M. R. & Mitchison, H. M. Sperm defects in primary ciliary dyskinesia and related causes of male infertility. *Cellular and molecular life sciences : CMLS* **77**, 2029-2048, doi:10.1007/s00018-019-03389-7 (2020).

35 Sui, W. *et al.* CCDC40 mutation as a cause of primary ciliary dyskinesia: a case report and review of literature. *Clin Respir J* **10**, 614-621, doi:10.1111/crj.12268 (2016).

36 Pasek, R. C. *et al.* Coiled-coil domain containing 42 (Ccdc42) is necessary for proper sperm development and male fertility in the mouse. *Developmental biology* **412**, 208-218, doi:10.1016/j.ydbio.2016.01.042 (2016).

37 Geng, Q. *et al.* A Novel Testis-Specific Gene, Ccdc136, Is Required for Acrosome Formation and Fertilization in Mice. *Reproductive sciences (Thousand Oaks, Calif.)* **23**, 1387-1396, doi:10.1177/1933719116641762 (2016).

38 Courtot, A. M. Presence and localization of the 60 KD calicin in human spermatozoa presenting postacrosomal sheath defects: preliminary results. *Molecular reproduction and development* **28**, 272-279, doi:10.1002/mrd.1080280309 (1991).

39 Longo, F. J., Krohne, G. & Franke, W. W. Basic proteins of the perinuclear theca of mammalian spermatozoa and spermatids: a novel class of cytoskeletal elements. *J Cell Biol* **105**, 1105-1120, doi:10.1083/jcb.105.3.1105 (1987).

40 Paranko, J., Longo, F., Potts, J., Krohne, G. & Franke, W. W. Widespread occurrence of calicin, a basic cytoskeletal protein of sperm cells, in diverse mammalian species. *Differentiation* **38**, 21-27, doi:10.1111/j.1432-0436.1988.tb00587.x (1988).

41 von Bülow, M., Heid, H., Hess, H. & Franke, W. W. Molecular nature of calicin, a major basic protein of the mammalian sperm head cytoskeleton. *Exp Cell Res* **219**, 407-413, doi:10.1006/excr.1995.1246 (1995).

42 He, X. *et al.* Novel homozygous CFAP69 mutations in humans and mice cause severe asthenoteratospermia with multiple morphological abnormalities of the sperm flagella. *J Med Genet* **56**, 96-103, doi:10.1136/jmedgenet-2018-105486 (2019).

43 Tang, S. *et al.* Biallelic Mutations in CFAP43 and CFAP44 Cause Male Infertility with Multiple Morphological Abnormalities of the Sperm Flagella. *Am J Hum Genet* **100**, 854-864, doi:10.1016/j.ajhg.2017.04.012 (2017).

44 Wu, H. *et al.* NovelCFAP43 andCFAP44 mutations cause male infertility with multiple morphological abnormalities of the sperm flagella (MMAF). *Reproductive biomedicine online* **38**, 769-778, doi:10.1016/j.rbmo.2018.12.037 (2019).

45 Chen, Y. F. *et al.* Cisd2 deficiency drives premature aging and causes mitochondria-mediated defects in mice. *Genes Dev* **23**, 1183-1194, doi:10.1101/gad.1779509 (2009).

46 Ishisaki, Z., Takaishi, M., Furuta, I. & Huh, N. Calmin, a protein with calponin homology and transmembrane domains expressed in maturing spermatogenic cells. *Genomics* **74**, 172-179, doi:10.1006/geno.2001.6544 (2001).

47 Davalieva, K. *et al.* Proteomic analysis of seminal plasma in men with different spermatogenic impairment. *Andrologia* **44**, 256-264, doi:10.1111/j.1439-0272.2012.01275.x (2012).

48 Pilch, B. & Mann, M. Large-scale and high-confidence proteomic analysis of human seminal plasma. *Genome biology* **7**, R40, doi:10.1186/gb-2006-7-5-r40 (2006).

49 Samanta, L., Parida, R., Dias, T. R. & Agarwal, A. The enigmatic seminal plasma: a proteomics insight from ejaculation to fertilization. *Reproductive biology and endocrinology : RB&E* **16**, 41, doi:10.1186/s12958-018-0358-6 (2018).

50 Dorin, J. R. & Barratt, C. L. Importance of β-defensins in sperm function. *Molecular human reproduction* **20**, 821-826, doi:10.1093/molehr/gau050 (2014).

51 Endo, S., Miyagi, N., Matsunaga, T., Hara, A. & Ikari, A. Human dehydrogenase/reductase (SDR family) member 11 is a novel type of 17β-hydroxysteroid dehydrogenase. *Biochemical and biophysical research communications* **472**, 231-236, doi:10.1016/j.bbrc.2016.01.190 (2016).

52 Hucho, F. The pyruvate dehydrogenase multienzyme complex. *Angew Chem Int Ed Engl* **14**, 591-601, doi:10.1002/anie.197505911 (1975).

53 Inaba, K. Molecular architecture of the sperm flagella: molecules for motility and signaling. *Zoolog Sci* **20**, 1043-1056, doi:10.2108/zsj.20.1043 (2003).

54 Inaba, K. Sperm flagella: comparative and phylogenetic perspectives of protein components. *Molecular human reproduction* **17**, 524-538, doi:10.1093/molehr/gar034 (2011).

55 Lindemann, C. B. & Lesich, K. A. Functional anatomy of the mammalian sperm flagellum. *Cytoskeleton (Hoboken)* **73**, 652-669, doi:10.1002/cm.21338 (2016).

56 Ha, S., Lindsay, A. M., Timms, A. E. & Beier, D. R. Mutations in Dnaaf1 and Lrrc48 Cause Hydrocephalus, Laterality Defects, and Sinusitis in Mice. *G3 (Bethesda)* **6**, 2479-2487, doi:10.1534/g3.116.030791 (2016).

57 Morohoshi, A. *et al.* Nexin-Dynein regulatory complex component DRC7 but not FBXL13 is required for sperm flagellum formation and male fertility in mice. *PLoS Genet* **16**, e1008585, doi:10.1371/journal.pgen.1008585 (2020).

58 Ikeda, T. *et al.* The mouse ortholog of EFHC1 implicated in juvenile myoclonic epilepsy is an axonemal protein widely conserved among organisms with motile cilia and flagella. *FEBS letters* **579**, 819-822, doi:10.1016/j.febslet.2004.12.070 (2005).

59 Nomikos, M. *et al.* Essential Role of the EF-hand Domain in Targeting Sperm Phospholipase Cζ to Membrane Phosphatidylinositol 4,5-Bisphosphate (PIP2). *The Journal of biological chemistry* **290**, 29519-29530, doi:10.1074/jbc.M115.658443 (2015).

60 Sutton, K. A. *et al.* Enkurin is a novel calmodulin and TRPC channel binding protein in sperm. *Developmental biology* **274**, 426-435, doi:10.1016/j.ydbio.2004.07.031 (2004).

61 Tsai, M. H. & Saier, M. H., Jr. Phylogenetic characterization of the ubiquitous electron transfer flavoprotein families ETF-alpha and ETF-beta. *Res Microbiol* **146**, 397-404, doi:10.1016/0923-2508(96)80285-3 (1995).

62 de Mateo, S., Castillo, J., Estanyol, J. M., Ballescà, J. L. & Oliva, R. Proteomic characterization of the human sperm nucleus. *Proteomics* **11**, 2714-2726, doi:10.1002/pmic.201000799 (2011).

63 Frapsauce, C. *et al.* Proteomic identification of target proteins in normal but nonfertilizing sperm. *Fertility and sterility* **102**, 372-380, doi:10.1016/j.fertnstert.2014.04.039 (2014).

64 Lehti, M. S., Kotaja, N. & Sironen, A. KIF3A is essential for sperm tail formation and manchette function. *Mol Cell Endocrinol* **377**, 44-55, doi:10.1016/j.mce.2013.06.030 (2013).

65 Chiu, W. W. & Chamley, L. W. Human seminal plasma antibody-binding proteins. *American journal of reproductive immunology (New York, N.Y. : 1989)* **50**, 196-201, doi:10.1034/j.1600-0897.2003.00067.x (2003).

66 Kamada, M., Liang, Z. & Koide, S. S. Identification of IgG and Fc-binding proteins in human seminal plasma and sperm. *Archives of andrology* **27**, 1-7, doi:10.3109/01485019108987645 (1991).

67 Witkin, S. S., Richards, J. M., Bongiovanni, A. M. & Zelikovsky, G. An IgG-Fc binding protein in seminal fluid. *American journal of reproductive immunology (New York, N.Y. : 1989)* **3**, 23-27, doi:10.1111/j.1600-0897.1983.tb00207.x (1983).

68 Chen, Y. *et al.* Glycerol kinase-like proteins cooperate with Pld6 in regulating sperm mitochondrial sheath formation and male fertility. *Cell Discov* **3**, 17030, doi:10.1038/celldisc.2017.30 (2017).

69 Asghari, A., Marashi, S. A. & Ansari-Pour, N. A sperm-specific proteome-scale metabolic network model identifies non-glycolytic genes for energy deficiency in asthenozoospermia. *Systems biology in reproductive medicine* **63**, 100-112, doi:10.1080/19396368.2016.1263367 (2017).

70 Khatchadourian, K. *et al.* Structural abnormalities in spermatids together with reduced sperm counts and motility underlie the reproductive defect in HIP1-/- mice. *Molecular reproduction and development* **74**, 341-359, doi:10.1002/mrd.20564 (2007).

71 Rao, D. S. *et al.* Huntingtin interacting protein 1 Is a clathrin coat binding protein required for differentiation of late spermatogenic progenitors. *Molecular and cellular biology* **21**, 7796-7806, doi:10.1128/mcb.21.22.7796-7806.2001 (2001).

72 Kamaruddin, M., Kroetsch, T., Basrur, P. K., Hansen, P. J. & King, W. A. Immunolocalization of heat shock protein 70 in bovine spermatozoa. *Andrologia* **36**, 327-334, doi:10.1111/j.1439-0272.2004.00629.x (2004).

73 Spinaci, M. *et al.* Immunolocalization of heat shock protein 70 (Hsp 70) in boar spermatozoa and its role during fertilization. *Molecular reproduction and development* **72**, 534-541, doi:10.1002/mrd.20367 (2005).

74 Volpe, S. *et al.* Comparative immunolocalization of heat shock proteins (Hsp)-60, -70, -90 in boar, stallion, dog and cat spermatozoa. *Reproduction in domestic animals = Zuchthygiene* **43**, 385-392, doi:10.1111/j.1439-0531.2007.00918.x (2008).

75 Olbrich, H. *et al.* Recessive HYDIN mutations cause primary ciliary dyskinesia without randomization of left-right body asymmetry. *Am J Hum Genet* **91**, 672-684, doi:10.1016/j.ajhg.2012.08.016 (2012).

76 Signorelli, J., Diaz, E. S. & Morales, P. Kinases, phosphatases and proteases during sperm capacitation. *Cell Tissue Res* **349**, 765-782, doi:10.1007/s00441-012-1370-3 (2012).

77 Feng, Y., Madungwe, N. B. & Bopassa, J. C. Mitochondrial inner membrane protein, Mic60/mitofilin in mammalian organ protection. *Journal of cellular physiology* **234**, 3383-3393, doi:10.1002/jcp.27314 (2019).

78 Bähler, M. & Rhoads, A. Calmodulin signaling via the IQ motif. *FEBS letters* **513**, 107-113, doi:10.1016/s0014-5793(01)03239-2 (2002).

79 Chauvin, T. R. & Griswold, M. D. Characterization of the expression and regulation of genes necessary for myo-inositol biosynthesis and transport in the seminiferous epithelium. *Biology of reproduction* **70**, 744-751, doi:10.1095/biolreprod.103.022731 (2004).

80 Clausen, J. Lactate dehydrogenase isoenzymes of sperm cells and tests. *The Biochemical journal* **111**, 207-218, doi:10.1042/bj1110207 (1969).

81 Guo, Y. *et al.* Proteomics analysis of asthenozoospermia and identification of glucose-6-phosphate isomerase as an important enzyme for sperm motility. *Journal of proteomics* **208**, 103478, doi:10.1016/j.jprot.2019.103478 (2019).

82 Saraswat, M. *et al.* Human Spermatozoa Quantitative Proteomic Signature Classifies Normo- and Asthenozoospermia. *Molecular & cellular proteomics : MCP* **16**, 57-72, doi:10.1074/mcp.M116.061028 (2017).

83 Baumgartner, M. R. *et al.* The molecular basis of human 3-methylcrotonyl-CoA carboxylase deficiency. *J Clin Invest* **107**, 495-504, doi:10.1172/jci11948 (2001).

84 Obata, K. *et al.* Human biotin-containing subunit of 3-methylcrotonyl-CoA carboxylase gene (MCCA): cDNA sequence, genomic organization, localization to chromosomal band 3q27, and expression. *Genomics* **72**, 145-152, doi:10.1006/geno.2000.6366 (2001).

85 Storey, B. T. & Kayne, F. J. Energy metabolism of spermatozoa. VII. Interactions between lactate, pyruvate and malate as oxidative substrates for rabbit sperm mitochondria. *Biology of reproduction* **18**, 527-536, doi:10.1095/biolreprod18.4.527 (1978).

86 Mera, P. E. & Escalante-Semerena, J. C. Multiple roles of ATP:cob(I)alamin adenosyltransferases in the conversion of B12 to coenzyme B12. *Appl Microbiol Biotechnol* **88**, 41-48, doi:10.1007/s00253-010-2773-2 (2010).

87 Kobayashi, T., Kaji, K. & Sakai, H. Nucleoside diphosphate kinase and the flagellar movement of glycerinated spermatozoa. *Journal of biochemistry* **79**, 413-418, doi:10.1093/oxfordjournals.jbchem.a131084 (1976).

88 Patel-King, R. S., Gorbatyuk, O., Takebe, S. & King, S. M. Flagellar radial spokes contain a Ca2+-stimulated nucleoside diphosphate kinase. *Molecular biology of the cell* **15**, 3891-3902, doi:10.1091/mbc.e04-04-0352 (2004).

89 Pei, J. & Grishin, N. V. Expansion of divergent SEA domains in cell surface proteins and nucleoporin 54. *Protein Sci* **26**, 617-630, doi:10.1002/pro.3096 (2017).

90 Haidl, G., Becker, A. & Henkel, R. Poor development of outer dense fibers as a major cause of tail abnormalities in the spermatozoa of asthenoteratozoospermic men. *Human reproduction (Oxford, England)* **6**, 1431-1438, doi:10.1093/oxfordjournals.humrep.a137283 (1991).

91 Rivkin, E., Tres, L. L. & Kierszenbaum, A. L. Genomic origin, processing and developmental expression of testicular outer dense fiber 2 (ODF2) transcripts and a novel nucleolar localization of ODF2 protein. *Molecular reproduction and development* **75**, 1591-1606, doi:10.1002/mrd.20911 (2008).

92 Lorenzetti, D., Bishop, C. E. & Justice, M. J. Deletion of the Parkin coregulated gene causes male sterility in the quaking(viable) mouse mutant. *Proceedings of the National Academy of Sciences of the United States of America* **101**, 8402-8407, doi:10.1073/pnas.0401832101 (2004).

93 Li, W. *et al.* A MEIG1/PACRG complex in the manchette is essential for building the sperm flagella. *Development (Cambridge, England)* **142**, 921-930, doi:10.1242/dev.119834 (2015).

94 Chavous, D. A., Hake, L. E., Lynch, R. J. & O'Connor, C. M. Translation of a unique transcript for protein isoaspartyl methyltransferase in haploid spermatids: implications for protein storage and repair. *Molecular reproduction and development* **56**, 139-144, doi:10.1002/(sici)1098-2795(200006)56:2<139::Aid-mrd3>3.0.Co;2-0 (2000).

95 O'Connor, C. M., Germain, B. J., Guthrie, K. M., Aswad, D. W. & Millette, C. F. Protein carboxyl methyltransferase activity specific for age-modified aspartyl residues in mouse testes and ovaries: evidence for translation during spermiogenesis. *Gamete research* **22**, 307-319, doi:10.1002/mrd.1120220308 (1989).

96 Siva, A. B. *et al.* Inhibiting sperm pyruvate dehydrogenase complex and its E3 subunit, dihydrolipoamide dehydrogenase affects fertilization in Syrian hamsters. *PloS one* **9**, e97916, doi:10.1371/journal.pone.0097916 (2014).

97 An, L. P. *et al.* Purification, molecular cloning and functional characterization of swine phosphatidylethanolamine-binding protein 4 from seminal plasma. *Biochemical and biophysical research communications* **423**, 690-696, doi:10.1016/j.bbrc.2012.06.016 (2012).

98 Kurokawa, M. *et al.* Functional, biochemical, and chromatographic characterization of the complete [Ca2+]i oscillation-inducing activity of porcine sperm. *Developmental biology* **285**, 376-392, doi:10.1016/j.ydbio.2005.06.029 (2005).

99 Nomikos, M., Kashir, J. & Lai, F. A. The role and mechanism of action of sperm PLC-zeta in mammalian fertilisation. *The Biochemical journal* **474**, 3659-3673, doi:10.1042/bcj20160521 (2017).

100 Saunders, C. M. *et al.* PLC zeta: a sperm-specific trigger of Ca(2+) oscillations in eggs and embryo development. *Development (Cambridge, England)* **129**, 3533-3544 (2002).

101 Gupta, G. S. in *Proteomics of Spermatogenesis* (ed G. S. Gupta) Ch. 20, 493-512 (Springer, 2005).

102 Han, Y., Haines, C. J. & Feng, H. L. Role(s) of the serine/threonine protein phosphatase 1 on mammalian sperm motility. *Archives of andrology* **53**, 169-177, doi:10.1080/01485010701314032 (2007).

103 Sasaki, M. *et al.* Disruption of the mouse protein Ser/Thr phosphatase 2Cbeta gene leads to early pre-implantation lethality. *Mech Dev* **124**, 489-499, doi:10.1016/j.mod.2007.04.001 (2007).

104 Sawada, H., Mino, M. & Akasaka, M. Sperm proteases and extracellular ubiquitin-proteasome system involved in fertilization of ascidians and sea urchins. *Advances in experimental medicine and biology* **759**, 1-11, doi:10.1007/978-1-4939-0817-2_1 (2014).

105 Sutovsky, P. Sperm proteasome and fertilization. *Reproduction (Cambridge, England)* **142**, 1-14, doi:10.1530/rep-11-0041 (2011).

106 Zimmerman, S. & Sutovsky, P. The sperm proteasome during sperm capacitation and fertilization. *Journal of reproductive immunology* **83**, 19-25, doi:10.1016/j.jri.2009.07.006 (2009).

107 Zimmerman, S. W. *et al.* Sperm proteasomes degrade sperm receptor on the egg zona pellucida during mammalian fertilization. *PloS one* **6**, e17256, doi:10.1371/journal.pone.0017256 (2011).

108 Chao, Y. *et al.* Structure and mechanism of the phosphotyrosyl phosphatase activator. *Molecular cell* **23**, 535-546, doi:10.1016/j.molcel.2006.07.027 (2006).

109 Batova, I. N., Petrov, M. G., Kyurkchiev, S. D. & Kehayov, I. R. Characterization of a sperm nuclear protein. *American journal of reproductive immunology (New York, N.Y. : 1989)* **36**, 49-57, doi:10.1111/j.1600-0897.1996.tb00138.x (1996).

110 Kwon, W. S. *et al.* A comprehensive proteomic approach to identifying capacitation related proteins in boar spermatozoa. *BMC Genomics* **15**, 897, doi:10.1186/1471-2164-15-897 (2014).

111 Mountjoy, J. R., Xu, W., McLeod, D., Hyndman, D. & Oko, R. RAB2A: a major subacrosomal protein of bovine spermatozoa implicated in acrosomal biogenesis. *Biology of reproduction* **79**, 223-232, doi:10.1095/biolreprod.107.065060 (2008).

112 Hu, Y. *et al.* RGS22, a novel testis-specific regulator of G-protein signaling involved in human and mouse spermiogenesis along with GNA12/13 subunits. *Biology of reproduction* **79**, 1021-1029, doi:10.1095/biolreprod.107.067504 (2008).

113 Cheng, G. Z., Li, J. Y., Li, F., Wang, H. Y. & Shi, G. X. Human ribonuclease 9, a member of ribonuclease A superfamily, specifically expressed in epididymis, is a novel sperm-binding protein. *Asian journal of andrology* **11**, 240-251, doi:10.1038/aja.2008.30 (2009).

114 Westmuckett, A. D. *et al.* Impaired sperm maturation in RNASE9 knockout mice. *Biology of reproduction* **90**, 120, doi:10.1095/biolreprod.113.116863 (2014).

115 Leahy, T., Rickard, J. P., Pini, T., Gadella, B. M. & de Graaf, S. P. Quantitative Proteomic Analysis of Seminal Plasma, Sperm Membrane Proteins, and Seminal Extracellular Vesicles Suggests Vesicular Mechanisms Aid in the Removal and Addition of Proteins to the Ram Sperm Membrane. *Proteomics* **20**, e1900289, doi:10.1002/pmic.201900289 (2020).

116 Dacheux, D. *et al.* Human FAM154A (SAXO1) is a microtubule-stabilizing protein specific to cilia and related structures. *Journal of cell science* **128**, 1294-1307, doi:10.1242/jcs.155143 (2015).

117 Sharma, R. *et al.* Proteomic analysis of human spermatozoa proteins with oxidative stress. *Reproductive biology and endocrinology : RB&E* **11**, 48, doi:10.1186/1477-7827-11-48 (2013).

118 Mi, Y., Shi, Z. & Li, J. Spata19 is critical for sperm mitochondrial function and male fertility. *Molecular reproduction and development* **82**, 907-913, doi:10.1002/mrd.22536 (2015).

119 Bégin, S., Bérubé, B., Boué, F. & Sullivan, R. Comparative immunoreactivity of mouse and hamster sperm proteins recognized by an anti-P26h hamster sperm protein. *Molecular reproduction and development* **41**, 249-256, doi:10.1002/mrd.1080410216 (1995).

120 Bérubé, B. & Sullivan, R. Inhibition of in vivo fertilization by active immunization of male hamsters against a 26-kDa sperm glycoprotein. *Biology of reproduction* **51**, 1255-1263, doi:10.1095/biolreprod51.6.1255 (1994).

121 Boué, F., Bérubé, B., De Lamirande, E., Gagnon, C. & Sullivan, R. Human sperm-zona pellucida interaction is inhibited by an antiserum against a hamster sperm protein. *Biology of reproduction* **51**, 577-587, doi:10.1095/biolreprod51.4.577 (1994).

122 Boué, F., Blais, J. & Sullivan, R. Surface localization of P34H an epididymal protein, during maturation, capacitation, and acrosome reaction of human spermatozoa. *Biology of reproduction* **54**, 1009-1017, doi:10.1095/biolreprod54.5.1009 (1996).

123 Frenette, G. & Sullivan, R. Prostasome-like particles are involved in the transfer of P25b from the bovine epididymal fluid to the sperm surface. *Molecular reproduction and development* **59**, 115-121, doi:10.1002/mrd.1013 (2001).

124 Gaudreault, C., C, L. g., Bérubé, B. & Sullivan, R. Hamster sperm protein, p26h: a member of the short-chain dehydrogenase/reductase superfamily. *Biology of reproduction* **61**, 264-273, doi:10.1095/biolreprod61.1.264 (1999).

125 Légaré, C., Gaudreault, C., St-Jacques, S. & Sullivan, R. P34H sperm protein is preferentially expressed by the human corpus epididymidis. *Endocrinology* **140**, 3318-3327, doi:10.1210/endo.140.7.6791 (1999).

126 Lessard, C., Parent, S., Leclerc, P., Bailey, J. L. & Sullivan, R. Cryopreservation alters the levels of the bull sperm surface protein P25b. *Journal of andrology* **21**, 700-707 (2000).

127 Montfort, L., Frenette, G. & Sullivan, R. Sperm-zona pellucida interaction involves a carbonyl reductase activity in the hamster. *Molecular reproduction and development* **61**, 113-119, doi:10.1002/mrd.1137 (2002).

128 Ackermann, M., Kubitza, M., Hauska, G. & Piña, A. L. The vertebrate homologue of sulfide-quinone reductase in mammalian mitochondria. *Cell Tissue Res* **358**, 779-792, doi:10.1007/s00441-014-1983-9 (2014).

129 Hemati, A., Azarnia, M., Hossein Modarressi, M. & Rahimi, A. Obtaining and characterization of anti-testis monoclonal antibodies: Invaluable tools toward the identification of testis antigens involved in fertilization. *Hum Antibodies* **26**, 209-218, doi:10.3233/hab-180340 (2018).

130 Pini, T. *et al.* Obesity significantly alters the human sperm proteome, with potential implications for fertility. *Journal of assisted reproduction and genetics* **37**, 777-787, doi:10.1007/s10815-020-01707-8 (2020).

131 Nagdas, S. K., Smith, L., McNamara, A., Hernandez-Encarnacion, L. & Medina-Ortiz, I. Identification and characterization of a bovine sperm acrosomal matrix protein and its mechanism of interaction with acrosomal hydrolases. *Molecular and cellular biochemistry* **410**, 11-23, doi:10.1007/s11010-015-2534-8 (2015).

132 Roy, A., Lin, Y. N., Agno, J. E., DeMayo, F. J. & Matzuk, M. M. Tektin 3 is required for progressive sperm motility in mice. *Molecular reproduction and development* **76**, 453-459, doi:10.1002/mrd.20957 (2009).

133 Takiguchi, H. *et al.* Characterization and subcellular localization of Tektin 3 in rat spermatozoa. *Molecular reproduction and development* **78**, 611-620, doi:10.1002/mrd.21352 (2011).

134 Tsukamoto, M. *et al.* Translocation of Tektin 3 to the equatorial segment of heads in bull spermatozoa exposed to dibutyryl cAMP and calyculin A. *Molecular reproduction and development* **84**, 30-43, doi:10.1002/mrd.22763 (2017).

135 Park, S. *et al.* CRISPR/Cas9-mediated genome-edited mice reveal 10 testis-enriched genes are dispensable for male fecundity. *Biology of reproduction* **103**, 195-204, doi:10.1093/biolre/ioaa084 (2020).

136 Zhu, Z. *et al.* Spermatogenesis is normal in Tex33 knockout mice. *PeerJ* **8**, e9629, doi:10.7717/peerj.9629 (2020).

137 Nakashima, A. *et al.* A positive role of mammalian Tip41-like protein, TIPRL, in the amino-acid dependent mTORC1-signaling pathway through interaction with PP2A. *FEBS letters* **587**, 2924-2929, doi:10.1016/j.febslet.2013.07.027 (2013).

138 Melideo, S. L., Jackson, M. R. & Jorns, M. S. Biosynthesis of a central intermediate in hydrogen sulfide metabolism by a novel human sulfurtransferase and its yeast ortholog. *Biochemistry* **53**, 4739-4753, doi:10.1021/bi500650h (2014).

139 Peknicova, J. *et al.* Expression of beta-tubulin epitope in human sperm with pathological spermiogram. *Fertility and sterility* **88**, 1120-1128, doi:10.1016/j.fertnstert.2006.12.070 (2007).

140 Salvolini, E. *et al.* Involvement of sperm plasma membrane and cytoskeletal proteins in human male infertility. *Fertility and sterility* **99**, 697-704, doi:10.1016/j.fertnstert.2012.10.042 (2013).
