# Supplementary material for: Zinc is a master-regulator of sperm function associated with binding, motility, and metabolic modulation during porcine sperm capacitation
Source: Commun Biol. 2022 Jun 3;5:538. doi: 10.1038/s42003-022-03485-8 (PMC9166710; doi:10.1038/s42003-022-03485-8)
Supplement: Supplementary file 9 — Reporting Summary [file 42003_2022_3485_MOESM9_ESM.pdf]

nature portfolio

Corresponding author(s): Michal Zigo, Peter Sutovsky

Last updated by author(s): Oct 13, 2021

Reporting Summary

Nature Portfolio wishes to improve the reproducibility of the work that we publish. This form provides structure for consistency and transparency in reporting. For further information on Nature Portfolio policies, see our Editorial Policies and the Editorial Policy Checklist.

Statistics

For all statistical analyses, confirm that the following items are present in the figure legend, table legend, main text, or Methods section.

n/a	Confirmed	
☐	☒	The exact sample size (*n*) for each experimental group/condition, given as a discrete number and unit of measurement
☐	☒	A statement on whether measurements were taken from distinct samples or whether the same sample was measured repeatedly
☐	☒	The statistical test(s) used AND whether they are one- or two-sided<br>*Only common tests should be described solely by name; describe more complex techniques in the Methods section.*
☒	☐	A description of all covariates tested
☐	☒	A description of any assumptions or corrections, such as tests of normality and adjustment for multiple comparisons
☐	☒	A full description of the statistical parameters including central tendency (e.g. means) or other basic estimates (e.g. regression coefficient) AND variation (e.g. standard deviation) or associated estimates of uncertainty (e.g. confidence intervals)
☐	☒	For null hypothesis testing, the test statistic (e.g. *F*, *t*, *r*) with confidence intervals, effect sizes, degrees of freedom and *P* value noted<br>*Give P values as exact values whenever suitable.*
☒	☐	For Bayesian analysis, information on the choice of priors and Markov chain Monte Carlo settings
☒	☐	For hierarchical and complex designs, identification of the appropriate level for tests and full reporting of outcomes
☒	☐	Estimates of effect sizes (e.g. Cohen's *d*, Pearson's *r*), indicating how they were calculated

Our web collection on statistics for biologists contains articles on many of the points above.

Software and code

Policy information about availability of computer code

Data collection	No software was used.
Data analysis	Zinc-binding site prediction software was developed in-house available at doi:10.1109/BIBM47256.2019.8983252; volcano plot analysis was performed according to https://bioconductor.org/packages/release/bioc/html/EnhancedVolcano.html; and PANTHER analyses were performed according to doi.org/10.1093/nar/gkaa1106 using http://www.pantherdb.org

For manuscripts utilizing custom algorithms or software that are central to the research but not yet described in published literature, software must be made available to editors and reviewers. We strongly encourage code deposition in a community repository (e.g. GitHub). See the Nature Portfolio guidelines for submitting code & software for further information.

Data

Policy information about availability of data

All manuscripts must include a data availability statement. This statement should provide the following information, where applicable:

- Accession codes, unique identifiers, or web links for publicly available datasets
- A description of any restrictions on data availability
- For clinical datasets or third party data, please ensure that the statement adheres to our policy

All data are provided within the MS submission.

Field-specific reporting

Please select the one below that is the best fit for your research. If you are not sure, read the appropriate sections before making your selection.

☒ Life sciences ☐ Behavioural & social sciences ☐ Ecological, evolutionary & environmental sciences

For a reference copy of the document with all sections, see nature.com/documents/nr-reporting-summary-flat.pdf

Life sciences study design

All studies must disclose on these points even when the disclosure is negative.

Sample size	The study was conducted on a non-transgenic fertile boar used for routine in vitro fertilization trials with high blastocyst yield, collected on weekly basis. No sample size calculations were performed, instead 4 replicates were performed, including a pilot replicate and three working replicates that were used for statistical and bio-informatics analyses. In proteomics studies, three to six replicates are generally sufficient to generate reliable data with statistical soundness.
Data exclusions	As states higher, the first - pilot replicate was excluded from the analysis as the purpose of this one was to optimize extraction, affinity purification, and MS detection.
Replication	The whole experiment can be replicated infinite times. The results obtained in all three working replicates were consistent and therefore assumed replicable. No software/analytical measures were taken to verify the reproducibility of results.
Randomization	The same boar was collected for all 4 replicates.
Blinding	Blinding was not relevant for this study since we were not comparing different groups.

Reporting for specific materials, systems and methods

We require information from authors about some types of materials, experimental systems and methods used in many studies. Here, indicate whether each material, system or method listed is relevant to your study. If you are not sure if a list item applies to your research, read the appropriate section before selecting a response.

Materials & experimental systems

n/a	Involved in the study	
☐	☒	Antibodies
☒	☐	Eukaryotic cell lines
☒	☐	Palaeontology and archaeology
☐	☒	Animals and other organisms
☒	☐	Human research participants
☒	☐	Clinical data
☒	☐	Dual use research of concern

Methods

n/a	Involved in the study	
☐	☐	ChIP-seq
☐	☒	Flow cytometry
☐	☐	MRI-based neuroimaging

Antibodies

Antibodies used	Rabbit polyclonal anti-CA2 (cat # PA5-78897) and rabbit polyclonal anti-CCDC39 (cat # PA5-57243) antibodies were purchased from Invitrogen, Carlsbad, CA. Rabbit polyclonal antibody against CCIN, anti-PT60 was made in house (Oko and Maravei, 1994, doi:10.1095/biolreprod50.5.1000; Oko and Maravei, 1995, doi:10.1002/jemt.1070320605). Goat anti-rabbit IgG (cat # 31460), HRP secondary antibody was purchased from ThermoFisher Scientific, Rockford, IL, USA. Goat anti-rabbit IgG, FITC (cat # 62-6111) was purchased from Zymed, San Francisco, CA.
Validation	Commercial antibodies were validated by the manufacturer, in-house antibody validation is included in the references listed above.

Animals and other organisms

Policy information about studies involving animals; ARRIVE guidelines recommended for reporting animal research

Laboratory animals	Sus scrofa, American Yorkshire, male, 2 years old
Wild animals	The study did not involve wild animals.
Field-collected samples	The study did not involve samples collected from the field.

Ethics oversight | All studies involving vertebrate animals were completed under the strict guidance of an Animal Care and Use protocol approved by the Animal Care and Use Committee (ACUC) of the University of Missouri.

Note that full information on the approval of the study protocol must also be provided in the manuscript.

ChIP-seq

Data deposition

☐ Confirm that both raw and final processed data have been deposited in a public database such as GEO.

☐ Confirm that you have deposited or provided access to graph files (e.g. BED files) for the called peaks.

Data access links
May remain private before publication. | *For "Initial submission" or "Revised version" documents, provide reviewer access links. For your "Final submission" document, provide a link to the deposited data.*

Files in database submission | *Provide a list of all files available in the database submission.*

Genome browser session
(e.g. UCSC) | *Provide a link to an anonymized genome browser session for "Initial submission" and "Revised version" documents only, to enable peer review. Write "no longer applicable" for "Final submission" documents.*

Methodology

Replicates | *Describe the experimental replicates, specifying number, type and replicate agreement.*

Sequencing depth | *Describe the sequencing depth for each experiment, providing the total number of reads, uniquely mapped reads, length of reads and whether they were paired- or single-end.*

Antibodies | *Describe the antibodies used for the ChIP-seq experiments; as applicable, provide supplier name, catalog number, clone name, and lot number.*

Peak calling parameters | *Specify the command line program and parameters used for read mapping and peak calling, including the ChIP, control and index files used.*

Data quality | *Describe the methods used to ensure data quality in full detail, including how many peaks are at FDR 5% and above 5-fold enrichment.*

Software | *Describe the software used to collect and analyze the ChIP-seq data. For custom code that has been deposited into a community repository, provide accession details.*

Flow Cytometry

Plots

Confirm that:

☒ The axis labels state the marker and fluorochrome used (e.g. CD4-FITC).

☒ The axis scales are clearly visible. Include numbers along axes only for bottom left plot of group (a 'group' is an analysis of identical markers).

☒ All plots are contour plots with outliers or pseudocolor plots.

☒ A numerical value for number of cells or percentage (with statistics) is provided.

Methodology

Sample preparation | Ejaculated and in vitro capacitated boar spermatozoa were fixed in 2.0% formaldehyde in PBS and permeabilized in 0.1% TrX-100 in PBS (PBST). Approximately 50 million spermatozoa were blocked with PBST supplemented with 5% normal goat serum (NGS). All the antibodies used for flow cytometric studies were previously characterized. Primary antibodies used were as follows: anti-carbonic anhydrase 2 (CA2, 1:80 dilution; PA5-78897, Invitrogen), anti-coiled coil domain 39 (CCDC39, 1:100 dilution, PA5-57243, Invitrogen), and anti-calicin [CCIN/PT60; 1:50 dilution; (Oko and Maravei, 1994)] all diluted in PBST supplemented with 5% NGS. Primary antibodies were added to sperm sample tubes and incubated overnight at 4°C. For negative controls, non-immune rabbit sera of comparable globulin concentrations were used instead of primary antibodies and processed in the same fashion. The following morning, spermatozoa were washed twice with PBST with 1% NGS, and appropriate species-specific secondary antibody, goat anti-rabbit conjugated to fluorescein isothiocyanate (GAR-FITC, 1:200 dilution, Zymed) in PBST with 1% NGS was added and allowed to incubate for 40 min at room temperature. For acrosome integrity assessment, peanut agglutinin conjugated to Alexa Fluor 647 (PNA-AF647, 1:2,000 dilution; Molecular Probes) was used, and 4',6-Diamidino-2-Phenylindole Dilactate (DAPI), a DNA stain (1:1500 dilution; Molecular Probes) was used as a reference and nuclear contrast stain. Both PNA-AF647 and DAPI were mixed and coincubated with secondary antibodies. After incubation with secondary antibodies, spermatozoa were washed twice with PBST with 1% NGS.

Instrument | Amnis FlowSight Imaging Flow Cytometer (AMNIS Luminex Corporation, Austin, TX) as described previously (Kennedy et al., 2014, doi:10.1002/mrd.22309)

Software	The raw image data were acquired using INSPIRE® software (AMNIS Luminex Corporation, Austin, TX), and data were analyzed using IDEAS® software (Version 6.2.64.0; AMNIS Luminex Corporation, Austin, TX).
Cell population abundance	The abundance of relevant cell population is usually 60-80%, the rest is either non-fluorescent debris or sperm heads without a tail and vice versa.
Gating strategy	Single cells were gated applying two consecutive gates: 1. Area of Bright field/ Aspect ratio of Bright field, 2. Area of DAPI channel/ Aspect ratio of DAPI channel

☐ Tick this box to confirm that a figure exemplifying the gating strategy is provided in the Supplementary Information.

Magnetic resonance imaging

Experimental design

Design type	*Indicate task or resting state; event-related or block design.*
Design specifications	*Specify the number of blocks, trials or experimental units per session and/or subject, and specify the length of each trial or block (if trials are blocked) and interval between trials.*
Behavioral performance measures	*State number and/or type of variables recorded (e.g. correct button press, response time) and what statistics were used to establish that the subjects were performing the task as expected (e.g. mean, range, and/or standard deviation across subjects).*

Acquisition

Imaging type(s)	*Specify: functional, structural, diffusion, perfusion.*
Field strength	*Specify in Tesla*
Sequence & imaging parameters	*Specify the pulse sequence type (gradient echo, spin echo, etc.), imaging type (EPI, spiral, etc.), field of view, matrix size, slice thickness, orientation and TE/TR/flip angle.*
Area of acquisition	*State whether a whole brain scan was used OR define the area of acquisition, describing how the region was determined.*

Diffusion MRI ☐ Used ☒ Not used

Preprocessing

Preprocessing software	*Provide detail on software version and revision number and on specific parameters (model/functions, brain extraction, segmentation, smoothing kernel size, etc.).*
Normalization	*If data were normalized/standardized, describe the approach(es): specify linear or non-linear and define image types used for transformation OR indicate that data were not normalized and explain rationale for lack of normalization.*
Normalization template	*Describe the template used for normalization/transformation, specifying subject space or group standardized space (e.g. original Talairach, MNI305, ICBM152) OR indicate that the data were not normalized.*
Noise and artifact removal	*Describe your procedure(s) for artifact and structured noise removal, specifying motion parameters, tissue signals and physiological signals (heart rate, respiration).*
Volume censoring	*Define your software and/or method and criteria for volume censoring, and state the extent of such censoring.*

Statistical modeling & inference

Model type and settings	*Specify type (mass univariate, multivariate, RSA, predictive, etc.) and describe essential details of the model at the first and second levels (e.g. fixed, random or mixed effects; drift or auto-correlation).*
Effect(s) tested	*Define precise effect in terms of the task or stimulus conditions instead of psychological concepts and indicate whether ANOVA or factorial designs were used.*

Specify type of analysis: ☐ Whole brain ☐ ROI-based ☐ Both

Statistic type for inference (See Eklund et al. 2016)	*Specify voxel-wise or cluster-wise and report all relevant parameters for cluster-wise methods.*
Correction	*Describe the type of correction and how it is obtained for multiple comparisons (e.g. FWE, FDR, permutation or Monte Carlo).*

Models & analysis

n/a	Involved in the study
☒	☐ Functional and/or effective connectivity
☒	☐ Graph analysis
☒	☐ Multivariate modeling or predictive analysis
